# Local protein detection with lateral flow assay read through tissue using X-ray excited luminescence chemical imaging

**DOI:** 10.1117/1.JBO.30.S2.S23915

**Published:** 2025-12-10

**Authors:** Yu Ding, K. Bradley Kelly, Morgan N. Reel, Matthew J. Case, Jeffrey N. Anker

**Affiliations:** a Clemson University, Department of Chemistry, Clemson, South Carolina, United States; b Clemson University, Department of Bioengineering and Medical Biophysics Program, South Carolina, United States

**Keywords:** X-ray scintillators, radioluminescence, immunoassay, implant-associated infection, implanted sensor

## Abstract

**Significance:**

Implant-associated infections can be difficult to detect at early stages. Noninvasively measuring inflammatory biomarkers such as C-reactive protein (CRP) in synovial fluid can improve early diagnosis and patient outcomes.

**Aim:**

We aim to show proof of principle for an implantable immunoassay device combined with an optical readout method to detect extracellular proteins through tissue. The system integrates lateral flow assay (LFA) strips with X-ray scintillators in a sealed implantable casing.

**Approach:**

An LFA strip is placed above a scintillator layer inside the implant. An X-ray beam excites the scintillator, generating luminescence that passes through the LFA. Test and control lines alter the transmitted light, and a fluorescent layer shifts the luminescence to red wavelengths to improve tissue penetration. External activation is enabled via high-intensity focused ultrasound to melt a wax seal.

**Results:**

We demonstrated protein detection using LFA strips for CRP and human chorionic gonadotropin through 6 mm of porcine muscle. Optical images clearly resolved 0.5-mm-wide test and control lines, with a strong correlation between signals measured with and without tissue. The device remained functional after extended fluid immersion.

**Conclusions:**

We show that LFA-based immunoassays can be integrated with X-ray excited luminescence imaging for through-tissue protein detection, advancing the development of implantable biosensors.

## 1 Introduction

Measuring local chemical concentrations through tissue is important for elucidating the biochemical basis of disease, as well as detecting and managing diseases. However, difficulties in noninvasively detecting proteins hinder these processes and make it challenging to personalize treatments. For example, over half of hospital-acquired infections are associated with implanted medical devices,^1^ and these infections often involve localized biofilms, which tolerate the host’s immune system and antibiotics. When infections are detected early, surgical irrigation and debridement with antibiotics are often successful; if not, devices such as prosthetic hips often need to be removed and temporarily replaced with antibiotic-impregnated spacers, which are in turn replaced in 1 to 6 months with a new device.^2^ These infections carry the risk of mortality and morbidity, and costs can be staggering.^3^ Detecting these infections can be challenging, especially when bacteria are localized near the implant surface at initial stages or during antibiotic treatment. Needle aspiration is commonly used to analyze the local fluid, but is contraindicated for repeated measurements of prosthetic joints and especially hips (which are performed by radiologists under fluoroscopy or ultrasound guidance) due to cost, pain, risk of dry taps (∼25%), and complications.^4^ Thus, noninvasive measurements are needed, which require implanted sensors to measure local protein concentrations near implanted medical devices. Unfortunately, most implanted biochemical sensors focus on enzymatically detected metabolites or redox-active neurotransmitters, not fully implanted extracellular protein sensors.^5^

Several noninvasive approaches can assist in the diagnosis of implant infection. First, symptoms including pain, swelling, fever, dehiscence, and wound leakage are indicative of infection; however, these are not always present, especially at early stages, and conversely, are often present in aseptic cases.^6^ Similarly, blood tests can detect infection and inflammation, but are not specific for implant infection. Noninvasive imaging includes computed tomography (CT) and magnetic resonance imaging (MRI) provide complementary information—CT delineates the extent of bone resorption around an implant, whereas MRI reveals soft-tissue abnormalities. However, both methods suffer from imaging artifacts near metal implants, and neither offers sufficiently reliable early detection of implant-associated infection, which is essential for effective treatment without implant removal and for reducing additional medical and surgical costs. Nuclear imaging techniques such as radiolabeled white blood cell scintigraphy are noninvasive and provide valuable diagnostic information for implant-associated infections.^7^ However, these methods are costly and time-consuming, requiring specialized facilities for blood cell tagging and imaging, which limits their availability for routine clinical use.^8^

Extracellular proteins are most commonly detected in laboratory settings using optical imaging techniques (e.g., specimens via ELISA, lateral flow assays (LFA),^9^ protein microarrays, and in tissue with optical fibers)^10^; however, to our knowledge, they have not previously been proposed for wireless/fiberless measurements through thick tissue. Three factors prevent light from acquiring high-resolution images through thick tissue to read out immunoassays: First, light exponentially attenuates as it propagates through tissue, which reduces signal intensity, especially for thick tissue and blue and green light. Second, overlying and adjacent tissue absorption and autofluorescence can obscure and overwhelm the sensor signal. Third, tissue scatters light, preventing it from maintaining focus. The light-attenuation and background challenges can be mitigated using red-exciting low-background techniques, such as long-lifetime phosphorescent or upconversion contrast agents. However, tissue typically has an optical scattering mean free path of ∼100  μm, and almost no unscattered photons are left to focus through several millimeters of tissue, preventing clear imaging of the fine lines and features that are found in complex analysis systems such as LFAs and gene chips.

**Fig. 1:**
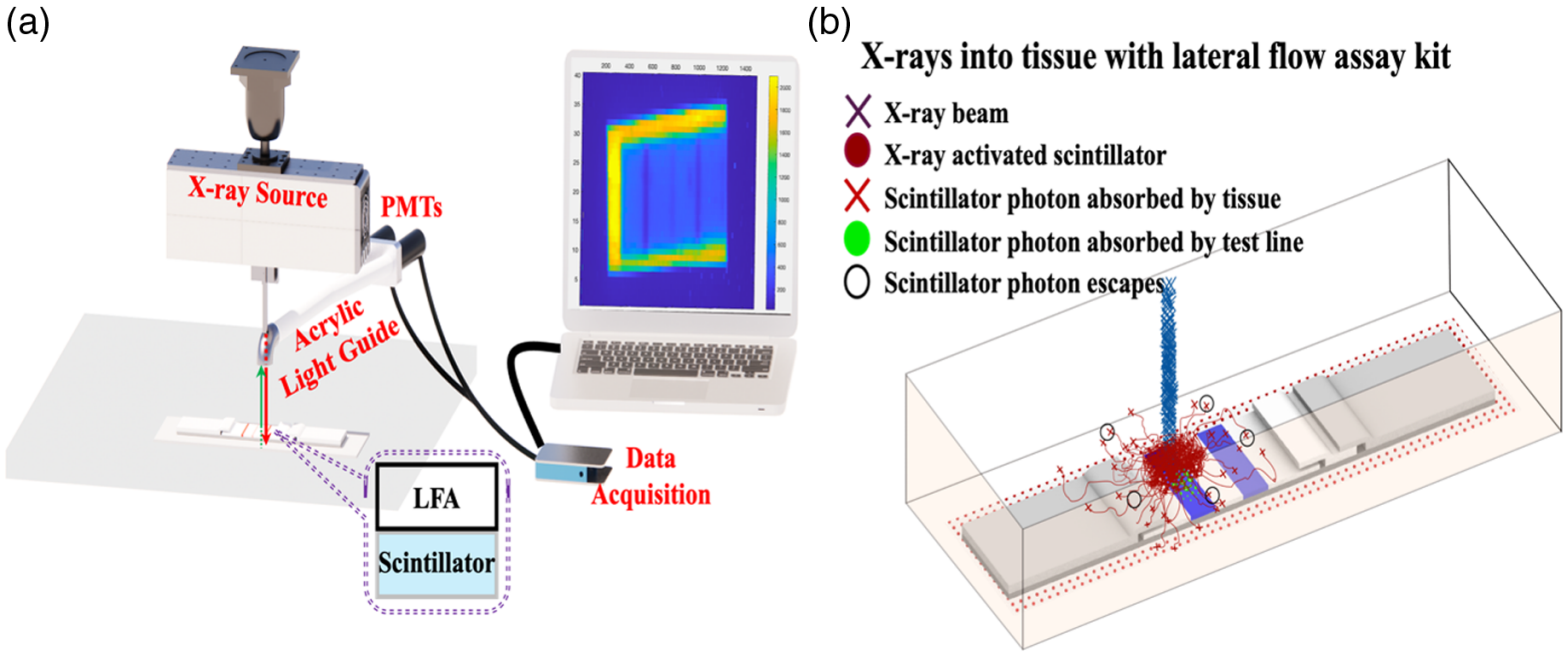
XELCI scanner setup and simulation. (a) Schematic of XELCI scanner setup to read a lateral flow assay. (b) Simulation of XELCI process: a focused X-ray beam (blue) irradiates scintillators, which generate visible light; some of this light is attenuated by the colored labels in the lateral flow strip, and the remaining transmitted light passes through tissue (with some absorption).

We developed X-ray Excited Luminescence Chemical Imaging (XELCI) for high-spatial resolution imaging of dyes through tissue. A focused X-ray beam excites a small spot of light on an implanted scintillator film, and the luminescence passes through a film containing indicator dyes, which modulate the intensity (Fig. 1). High-resolution images, limited by the X-ray beam size, are formed by measuring the transmitted light through the tissue at each X-ray beam position. Applications include imaging optical absorption from pH indicators on implanted devices in cadaveric tissue^11^ and live rabbits^12^ and detecting doxorubicin-loaded nanoparticles.^13^ Herein, we extend this approach and show proof-of-principle that a modified LFA can be read through tissue. A scintillator film is placed beneath the LFA absorptive lines, and the light passing through the assay strip and tissue is detected. Observing the test, reference, and control regions can also be extended in the future to other imaging or sensing methods based upon μ-LEDs,^14^ ultrasound modulation,^15^ magnetic modulation,^16^ or photoacoustic tomography.^17^ Although significant work will be required to make functional devices, we utilized commercial LFAs to show proof of principle for imaging and analysis through tissue.

## 2 Materials and Methods

### 2.1 Materials

Human chorionic gonadotropin (HCG) Rapid Detection Pregnancy Test was purchased from Clearblue (Vallejo, California, USA). CRP commercial LFA were purchased from IVT Imuno (Hradec Králové, Czech Republic). HCG was purchased from Thermo Fisher Scientific (Waltham, Massachusetts, USA). C-reactive protein (CRP) was purchased from Sigma-Aldrich (St Louis, Missouri, USA). Phosphate-buffered saline (PBS) was purchased from Sigma-Aldrich (St Louis, Missouri, USA). Bovine calf serum was purchased from Sigma-Aldrich (St Louis, Missouri, USA). X-ray scintillator microphosphors, ${\mathrm{Gd}}_{2}O_{2}S:\mathrm{Tb}$ (UKL63/N-R1), and ${\mathrm{Gd}}_{2}O_{2}S:\mathrm{Tb}$ (UKL65/N-R1) were purchased from Phosphor Technology Ltd. (Stevenage, UK). Polydimethylsiloxane (PDMS) base and its curing agent [2065622] were purchased from Ellsworth Adhesive (Germantown, Wisconsin, USA). Fluorescent plastic slides were provided by Chroma Technology (Bellows Falls, Vermont, USA). Porcine tissue (cutlets) was acquired from Walmart. PETG 1.75 mm Filament [M-1VF-5877] was purchased from Matter Hackers (Lake Forest, California, USA). Paraffin Wax (Laboratory Grade, 1 lb.) [879190] was purchased from Carolina Biological Supply (Burlington, North Carolina, USA), which has a melting point of 56°C.

### 2.2 XELCI Scanner Setup

Figure 1(a) shows the schematic of the XELCI setup, and Fig. 1(b) is the simulation of the XELCI process. X-ray is emitted by an X-ray source (iMOXS, Institute for Scientific Instruments GmbH, Berlin, Germany) and focused into a beam by a polycapillary lens (5-cm focal distance from capillary tip), which is direct to the surface of a x-y-z motorized stage (30×15×6  cm travel, Models: LTS300 and LTS150, Thorlabs Inc., Newton, New Jersey, USA for x- and y-axis and Motorized Linear Vertical Stage Model AT10-60, Motion Control, Smithtown, New York, USA for the z-stage). The X-ray beam intensity at the focus height (5 cm from the tip of the polycapillary) was previously determined by comparing the radiochromic film with the exposure calibration reference of the same film provided by the manufacturer.^12^ The dose depends on the scanning speed, step size, and duration of the scan; at a typical scan speed of 5 mm/s with a step size of 250  μm, the local absorbed dose was found to be 50 rad or 0.5 Gy. This value is well below the threshold dose associated with cutaneous radiation injury (radiation burns), which may develop following exposure to doses as low as ∼2  Gy.^18^ The 0.5 Gy dose represents the localized dose absorbed at the skin and will be much lower within the muscle tissue underneath the skin as we are using a relatively lower energy X-ray beam (50 keV).

The combined LFA strip and scintillator layer is placed on the top of the stage, and its position is adjusted beneath this X-ray beam by using crossed laser lines to show the focus spot. Most of the focused X-ray beam excites the scintillator particles, resulting in luminescence, and a small amount of the X-rays will be absorbed by tissue. After X-ray excitation, the scintillator emits visible luminescence. The light traverses the overlying tissue and the LFA strip; most photons are attenuated by absorption and scattering, and only a small fraction exits the sample. We collect the transmitted luminescence with either a liquid light guide (model 77638, Newport Corporation, Irvine, California, USA) or an in-house-machined acrylic light guide, depending on the LFA format. The light collected by either light guide is delivered to a photomultiplier tube (P25PC-16, SensTech, Surrey, UK) for detection (PMT; P25PC-16, SensTech, Surrey, UK). One collects all wavelengths, whereas the other has an optical filter to pass the 700-nm light and filter out 580- to 660-nm wavelengths. An orange filter (ROSCO Company, Roscolux, Supergel, R14 Medium Straw) placed before the entrance to the light guide further cuts out low wavelength light <460  nm before reaching either PMT.

To further illustrate the process, Fig. 1(b) shows a Monte Carlo simulation of the process of 20 keV X-ray photon transmittance through the tissue, generation of visible light in the scintillator, propagation of the visible photons through the tissue, and escape from the tissue surface. The simulation was done according to our previous study.^19^ It shows X-ray in a 250-μm diameter beam generally moving in straight lines through tissue, with some absorption and minimal scattering (based on NIST standard reference database 126 on X-ray mass attenuation coefficients), strong absorption by the ${\mathrm{Gd}}_{2}O_{2}S:\mathrm{Eu}$ scintillators, and strong absorption and scattering of light by the tissue ($\mu_{s}=1/100\;\mu m$; g=<cos(θ)≥0.95; $\mu_{a}=1/1.7\;\mathrm{cm}$). The number of photons making it through the tissue skin depends strongly on the initial absorption near the test line, especially as most of the light making it through the strip is reflected if tissue backscattering causes it to return. This provides high spatial resolution imaging of the local absorption near the test line, limited by the X-ray beam width and scintillator width rather than the diffuse scattering in the tissue.

To minimize the external light background, the entire setup is enclosed within a light-tight box. A data acquisition (DAQ) board (NI, cDAQ-9171, National Instruments, Austin, TX) is used to count the pulses from each PMT, which connects with the computer and uses a LabVIEW program (National Instruments, Austin, TX) to control the position of the stage, as well as to record PMT counts and stage position over time. In addition, the program provides a real-time display of acquired images on the computer screen. The scanning process and signal processing are explained in our previous work.^20^

### 2.3 Sealing LFA Against Liquid Exposure with Wax and Breaking the Seal with Focused Ultrasound to Start the LFA

We initially employed HCG LFA strips to evaluate the potential of using an ultrasound device to liquefy the Paraffin wax that filled the drilled hole on a pregnancy test, thus enabling water to enter the cap and initiate the LFA. There are many brands of HCG LFA tests, and we selected the Rapid Detection Pregnancy Test from Clearblue because the control and test lines on the LFA are blue colored, which can strongly attenuate the red light from scintillator film. As illustrated in Fig. S1a in the Supplementary Material, we modified the HCG LFA by drilling a hole halfway through the cap using a drill-bit machine. Then, a flat-sided tool was used as a barrier for the Paraffin wax when filling the hole, as demonstrated in Fig. S1b in the Supplementary Material. We weighed out 2.60 g of low-melting point paraffin wax and heated it in a 250-mL Erlenmeyer flask using a hot plate at 80°C for 10 min. Once melted, the liquid wax was poured into the drilled hole and allowed to cool and solidify. We used two HCG LFA with paraffin wax-filled caps to conduct the ultrasound test. To prevent water from getting into the tests, we wrapped the pregnancy tests with Parafilm, as depicted in Fig. S1c in the Supplementary Material. The setup for the ultrasound test is depicted in Fig. S2a in the Supplementary Material, where two pregnancy tests (one is the control and another is the experimental test) were held by a stand, and the extend rod was connected to the tests using Zip ties. A clearer photo is presented in Fig. S2b in the Supplementary Material. After these preparations were completed, we submerged the pregnancy tests beneath the water and connected the ultrasound source. As shown in Fig. S2c in the Supplementary Material, the ultrasound successfully caused the paraffin wax to melt for the assay on the right, allowing water to enter the cap and run the assay; because there was no HCG in the solution, the test yielded a negative result.

### 2.4 Fabrication of Scintillator Layer

The silicone elastomer and the curing agent were mixed at a 10:1 weight ratio. Then, 8.0-μm diameter ${\mathrm{Gd}}_{2}O_{2}S:\mathrm{Eu}$ X-ray scintillator microphosphors were added to that PDMS solution at a 4:1 weight ratio (4 g microphosphors with 1 g PDMS). The PDMS precursor with ${\mathrm{Gd}}_{2}O_{2}S:\mathrm{Eu}$ scintillator particles was mixed homogeneously and degassed using a vacuum desiccator and then cured the PDMS mixture in an oven at 100°C to form about 0.5-mm-thick ${\mathrm{Gd}}_{2}O_{2}S:\mathrm{Eu}$ scintillator-PDMS layer. The 0.5-mm-thick film was made using a reaction cell. This reaction cell was composed of a 0.5-mm-thick transparent silicone rubber film, which was cut into a rectangular shape with a rectangular hole in the middle as a spacer, and was sandwiched between two glass slides. The PDMS precursor was injected into this cell. The same procedures were repeated to use 10-μm-diameter of ${\mathrm{Gd}}_{2}O_{2}S:\mathrm{Tb}$ scintillator microphosphors to prepare 0.5-mm-thick ${\mathrm{Gd}}_{2}O_{2}S:\mathrm{Tb}$ scintillator-PDMS films. These two different layers were cut into the same shapes as the LFA strips.

### 2.5 HCG Assays

Five concentrations of HCG solutions (0, 7.5, 15, 62.5, and 125 mlU/mL) were prepared in bovine calf serum. A few drops of the prepared solutions were pipetted onto each HCG LFA strip for 5 min. We utilized a 3D printer to construct a sealed container using PETG 1.75 mm filament and left a small opening on the lid. We then placed our ${\mathrm{Gd}}_{2}O_{2}S:\mathrm{Eu}$ scintillator/PDMS film in this container, placed the HCG LFA strips above the scintillator film, and closed the container. The XELCI imaging was acquired from above the LFA layer using a liquid light guide as the light collector sending light to the photomultiplier tubes. Porcine muscle tissue was sliced into the desired thickness (6 mm) using an electric food slicer (model 630, Chef’s Choice, Avondate, Pennsylvania, USA). The slice was sealed in clear plastic wrap and positioned on the top of the container. XELCI was repeated with and without tissue for the five HCG concentrations by switching out the LFA in the LFA/scintillator film container.

### 2.6 CRP Assays

CRP solutions with concentrations of 0, 1, and 5 mg/L were prepared in PBS solution. A few drops of the prepared solution were pipetted onto each CRP LFA strip for 10 min. After the incubation, the sample pads, conjugated pads, and absorbent pads were cut off with scissors, leaving the nitrocellulose membranes to show control, reference, and test lines. The nitrocellulose membrane of each strip was placed on top of the ${\mathrm{Gd}}_{2}O_{2}S:\mathrm{Tb}$ scintillator. We improved the XELCI scanner prior to CRP imaging by replacing the liquid light guide with an acrylic light guide as the light collector and replicating the same procedures that were previously performed for HCG imaging. We initially obtained XELCI images using CRP LFA strips with different concentrations. Next, we placed a 6-mm-thick porcine tissue layer over the combined scintillator film-LFA device and imaged once more. A green exciting/red-emitting fluorescence slide was then placed between the porcine tissue and the device and imaged again using XELCI.

### 2.7 Image Reconstruction

A customized LabVIEW program managed the motorized stage and gathered data from both stage position and photon counts versus time for two PMTs. A customized MATLAB script processed the raw intensity and motor position versus time data into an intensity versus position image using a linear interpolation algorithm.

## 3 Results and Discussion

We first performed and analyzed data with an HCG-detecting LFA strip with blue colored lines on red-emitting radioluminescent film (Sec. 3.1). We then updated the XELCI scanner and ran a study with a CRP-detecting LFA strip with red-colored lines on a green-emitting radioluminescent film with and without the addition of a red fluorescent film (Sec. 3.2). We then show proof-of-principle for storing LFAs in wet environments and starting flow with an external trigger (Sec. 3.3) and discuss limitations and alternatives in Sec. 3.4.

### 3.1 HCG-Detecting LFA

**Fig. 2:**
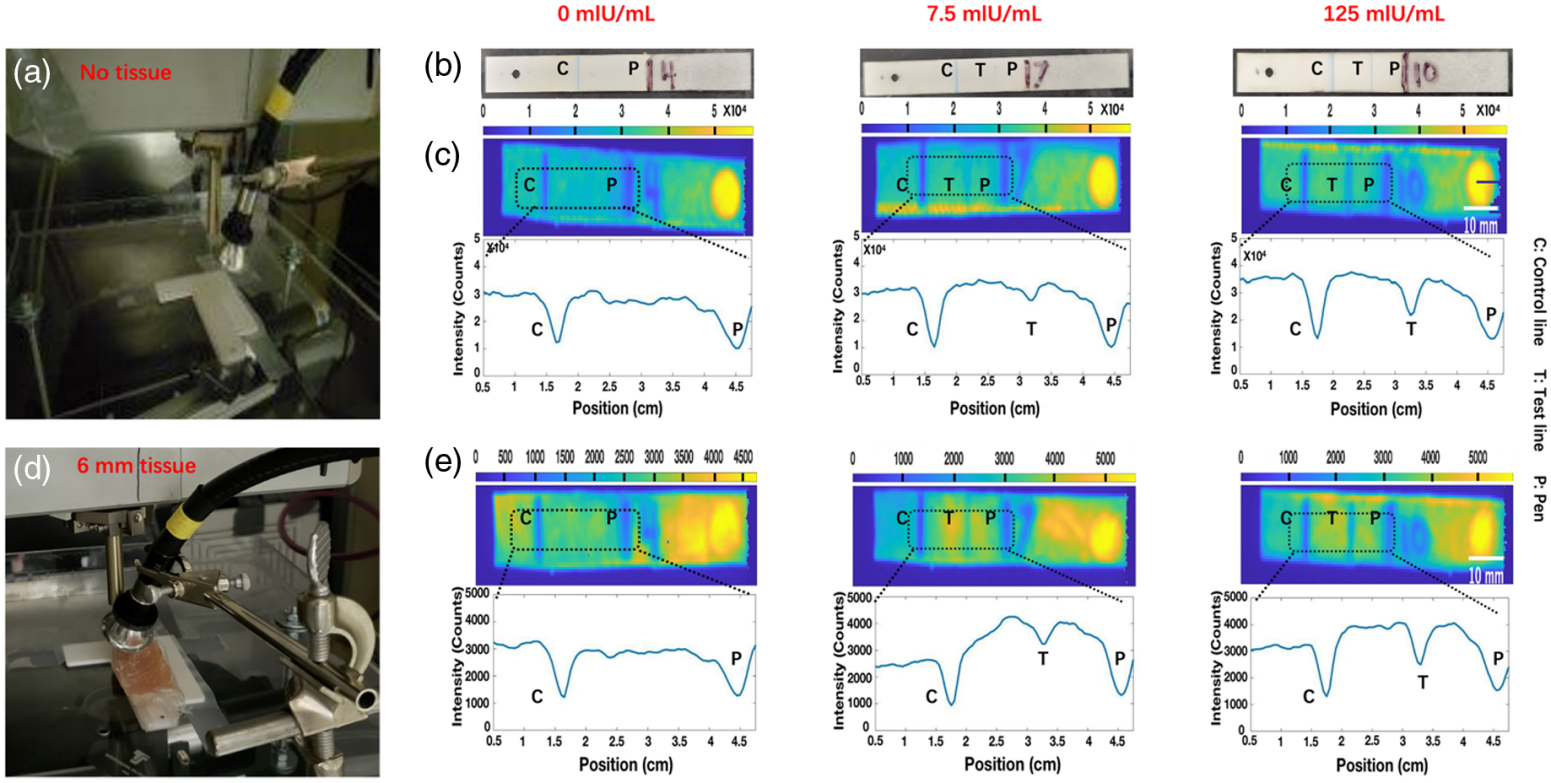
XELCI LFA assay setup and imaging with/without 6-mm chicken breast tissue. (a) Photo of HCG LFA strip and casing. Each casing has a small hole, a scintillator layer, a strip with control and test lines (though not developed at 0 mlU/mL sample), and a black line with the sample number written by a pen. (b) Photos of the test strip at 0, 7.5, and 125 mIU/mL. (c) XELCI intensity images of LFAs in casing exposed to various HCG concentrations. (d) Photo of HCG LFA and scintillator layer covered by 6-mm chicken breast tissue in the XELCI instrument. (e) XELCI images of the LFA strip in casing covered by chicken breast tissue with various HCG concentrations. Each assay was performed with n=1 trial per concentration.

The sensor consists of two components: a commercial LFA strip containing only the nitrocellulose membrane with the detection lines, and an X-ray scintillator layer, which is a PDMS film that homogeneously incorporates mostly red-emitting ${\mathrm{Gd}}_{2}O_{2}S:\mathrm{Eu}$ microphosphors or mostly green-emitting ${\mathrm{Gd}}_{2}O_{2}S:\mathrm{Tb}$ microphosphors. When the scintillator layer was irradiated by the X-ray, some of the luminescence was attenuated by the detection lines on the LFA strips. The intensity of this luminescence spectrum varies depending on the absorption of the test line, which depends on antigen concentration, whereas the control line is independent of antigen concentration (Fig. 2). When no antigen is present, the test line should have no more absorption than the rest of the strip; as antigen concentration increases, the test line should progressively attenuate more light, until the line intensity saturates.

The commercial HCG-detecting LFA used in this paper is a rapid test. According to the FDA’s 510(k) summary for the Clearblue Easy Pregnancy Test, data from 30 samples indicate a limit of detection cutoff concentration of ∼9 to 10 mIU/mL.^21^ For specificity, in their 510(k), Clearblue tested several common potential cross-reactants in HCG-negative solutions. Luteinizing hormone (LH) (up to 5000 mIU/mL) and thyroid-stimulating hormone (TSH) (up to 1.0 mIU/mL) did not interfere with the test. Although follicle-stimulating hormone (FSH) levels of 1000 and 5000 mIU/mL produced some false-positive results, 1000 mIU/mL exceeds normal physiological levels, and this cross-reactivity is not a concern. The working HCG range is therefore 10 to 500 mIU/mL.

We used red-emitting ${\mathrm{Gd}}_{2}O_{2}S:\mathrm{Eu}$ phosphors to illuminate the HCG LFA strips, which were attenuated by the blue-colored nanoparticle labels used in “Clear Blue” HCG LFA strips. The emission spectrum of ${\mathrm{Gd}}_{2}O_{2}S:\mathrm{Eu}$ phosphors is shown in Fig. S3 in the Supplementary Material. Figure 2(a) is a photo of the setup without tissue, and Fig. 2(b) is a series of photos of HCG LFA strips. Figure 2(c) shows XELCI images and corresponding line scans for LFA’s developed with varying HCG concentrations (0, 7.5, and 125 mIU/mL). The black rectangle on the XELCI images indicates the location of the line profile. The corresponding line profiles show a control line peak, a test line peak (except 0 mlU/mL), and a labeled number peak (written in ink that absorbed some scintillator luminescence). Figures 2(d) and 2(e) show the corresponding setup photo, XELCI images, and line profile acquired through 6 mm of porcine tissue. The images with and without tissue look very similar at all concentrations, although the absolute intensity is attenuated by about 10× through the tissue. At 0 mlU/mL HCG, only the control line is evident (both with and without tissue), whereas the test line intensity increases at higher concentrations. Figure S4 in the Supplementary Material presents a larger set of concentrations (0, 7.5, 15, 30, 62.5, and 125 mlU/mL). An important imaging metric is the pixel-to-pixel signal-to-noise ratio (SNR), based on a 20-μm pixel width, evaluated from individual and averaged line profiles. For the liquid light guide assay, we used the HCG sample (125 mIU/mL) without tissue as an example for analysis. The noise of baseline 1 was ∼19.5% for individual lines and around 5% for the average of n=17 lines. The acrylic light guide demonstrated higher optical collection efficiency; using the CRP 0 mg/L sample (without tissue) as an example, the noise of baseline 1 was about 2% for individual lines and 1% for the average of n=16 lines. The average drop between the peak and baseline at the control line (typically 1 mm wide) was ∼14%. In practice, the sensitivity is not limited by short-term noise—the observed dips are roughly 100 times larger than the expected signal at 1 mg/mL—but rather by drift and artifacts, likely caused by nonuniformity in the scintillator film or nitrocellulose strip. For example, in Fig. 5(d), the 5 mg/mL sample shows a spot near the target line that is about 1/5 the height of the target peak. This spot is clearly an artifact, as it appears in the wrong position, has an atypical horizontal width, and does not extend vertically across the strip, yet it is much larger than instrument noise and a potential source of false readings. Note, small variations are not clinically significant, as Parvizi’s group reported CRP levels increase an average of 20× (to 40 mg/L)^22^ during prosthetic joint infection; similarly, Balato’s group reported 7× increase to 22 mg/L.^23^

Data Analysis: To quantify the absorption peaks, we found the peak intensity and adjacent background regions as shown in Fig. S5 in the Supplementary Material. We used the CRP assay result as an example to illustrate absorption peak quantification in XELCI images because it only has the necessary components compared with HCG assays (mentioned in the later section). The logical approach applied in the HCG assay aligns similarly with that of the CRP assay. For the line profile, we selected the central 4-mm region of the strip on the image (image rows 15 to 27). We then calculated the mean baseline data 1, 2, 3, and 4 and found the peak data of the control, reference, and test line. All the mentioned data are in Table S1 in the Supplementary Material. Based on these data, we determined the proper calibration curves based on the intensity and concentration. We also processed other raw data from HCG and CRP experiments. The intensity plots are shown in Fig. S6 in the Supplementary Material. We found that these images did not have the same absolute intensity scale, and the values needed to be normalized. Thus, we utilized the transmittance and absorbance equations to calculate from the raw data. For each line (control or test), two baselines were placed on the left and right shoulders of the line profile and averaged to obtain the local baseline intensity $I_{\text{baseline}}$. A background region showing dark current away from the scintillator film was used to obtain $I_{\mathrm{bg}}$. The line peak intensity at the line center was recorded as $I_{\text{peak}}$. Transmittance for the control line was computed as

$$T_{\mathrm{Control}}=\frac{I_{\mathrm{control}\;\mathrm{peak}}-I_{\mathrm{bg}}}{I_{\mathrm{control}\;\mathrm{baseline}}-I_{\mathrm{bg}}}.$$

Absorbance-style values were then obtained as: $A_{\mathrm{cont}}=-\log_{10}(T_{\mathrm{Control}})$.

The same procedure was applied to the test line to compute the transmittance of the test, $A_{\text{test}}$.

**Fig. 3:**
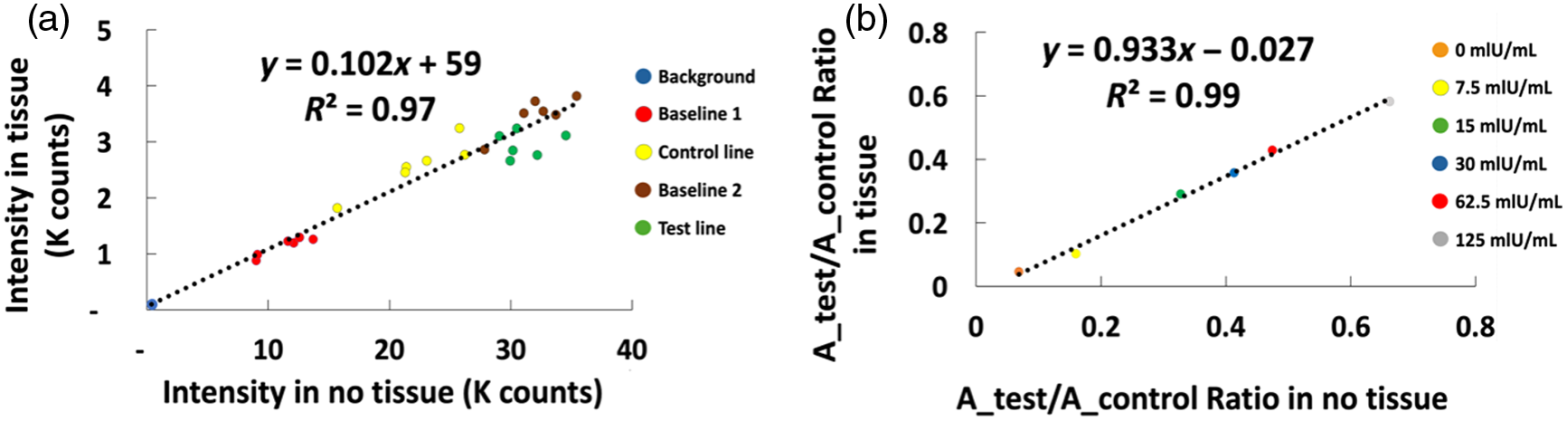
Correlation between HCG LFA data with and without tissue. (a) The intensity from all concentrations with tissue versus no tissue. (b) Ratio of test line absorbance/control line absorbance with versus without tissue. Both plots show strong agreement between measurements with and without tissue.

For a combination of kinetic and thermodynamic reaction reasons, we would expect the absorbance ratio of the test line to the control line absorbance to depend on concentration, approximately fitting a Langmuir isotherm equation: linear at low concentrations, and saturating at high concentrations.^24^ In addition, at very high antigen concentrations (typically ∼50  IU/mL), excess antigen would coat both the capture antibody on the test strip and the detection antibody on the nanoparticle label, reducing overall binding in the sandwich assay, a phenomenon called “the hook effect.”^25^ Although our results were quite variable at high concentrations and did not fit the expected curve (Fig. S7 in the Supplementary Material), we could clearly distinguish low levels of HCG clinically relevant in pregnancy for which the commercial was designed. It should be noted that the devices were designed for “yes/no” answers, not quantification, and that we read devices long after the typical “30-min” read window. Based on the HCG assay data (all components and all concentrations), we got a strong correlation ($R^{2}>0.96$) between measurements with versus without tissue, although the intensity through tissue was attenuated by about 10× [Fig. 3(a)]. We then calculated an effective peak absorbance for control and test regions from $-\log_{10}$ of the ratio of peak intensity to adjacent background. The ratio of test to control peak absorbance with and without tissue strongly correlated ($R^{2}>0.99$). The slope is 0.93 (close to 1) and indicates that measurements with and without tissue agreed well [Fig. 3(b)].

Resolution: To quantify transmittance, it is important that the image resolution is similar to, or better than, the width of the test lines so that the lines can be well resolved and distinguished from the background. In these XELCI images, 1 horizontal pixel represents 20  μm, and we found that imaging through tissue did not significantly broaden the control line (full width at half maximum intensity was 0.80 mm both with and without tissue). This is consistent with a sharp 80% to 20% knife-edge resolution compared with the peak width (measured from the left edge of the scintillator film, it was 420  μm without tissue versus 480  μm with tissue). Interestingly, this high resolution also allowed us to observe through tissue the numbers written in Sharpie pen on the test strip to label the trial (Fig. 2 and Fig. S4 in the Supplementary Material).

The HCG assay showed proof of principle for reading complex assays using a test that was widely available in pharmacies. Admittedly, the intended urine analysis format is easiest for determining pregnancy, and HCG concentration can also be measured in blood, so there is no compelling reason to implant an HCG sensor. Nonetheless, the general LFA platform can be easily adapted to other analytes that are useful to measure locally for preclinical research on local conditions such as detecting and monitoring implant infections and other local diseases.

### 3.2 CRP Assays

To show generality, we repeated the experiment with a CRP LFA. CRP is more relevant to local infection. Although it is a general blood inflammation biomarker, there are reports that local concentrations in synovial fluid are more accurate for prosthetic joint infection.^22^

The commercial CRP-detecting LFA used in this experiment is a semi-quantitative type, featuring a reference line between the control and test lines to indicate a cut-off concentration of 10 mg/L for CRP in blood after dilution in a preparation buffer (10× dilution). From 0 to 10 mg/L, only the control and reference lines show a clear signal. From 10 to 30 mg/L, all lines show a signal, but the test line displays a dim signal. Above 30 mg/L, all lines show a signal, with the test line showing a stronger signal. The product specification also claims high specificity and accuracy (>99%).^21^

The CRP-detecting experiments were performed using an upgraded scanner, and the experiments differed from the HCG-sensing experiments in several ways. First, we improved the XELCI scanner’s optical collection system by replacing the liquid light guide with a solid machined acrylic rod light guide in air and reflective optic, which together had a larger aperture and acceptance angle.^12^ Second, the CRP assay was a semi-quantitative LFA, with an additional reference line to show more information than the HCG-detecting LFA. Third, the LFA itself used gold nanoparticle labels, which absorbed green and blue light much more than red and near-infrared. Thus, to increase attenuation by red lines in CRP kits, we changed the scintillator particles from primarily red-emitting (620 nm) ${\mathrm{Gd}}_{2}O_{2}S:\mathrm{Eu}$ to primarily green-emitting (545 nm) ${\mathrm{Gd}}_{2}O_{2}S:\mathrm{Tb}$. Finally, to eliminate the influence of unbounded antibody-nanoparticle conjugates in conjugated pads and absorbent pads on later XELCI imaging, we only left the nitrocellulose membranes part containing control, reference, and test lines.

**Fig. 4:**
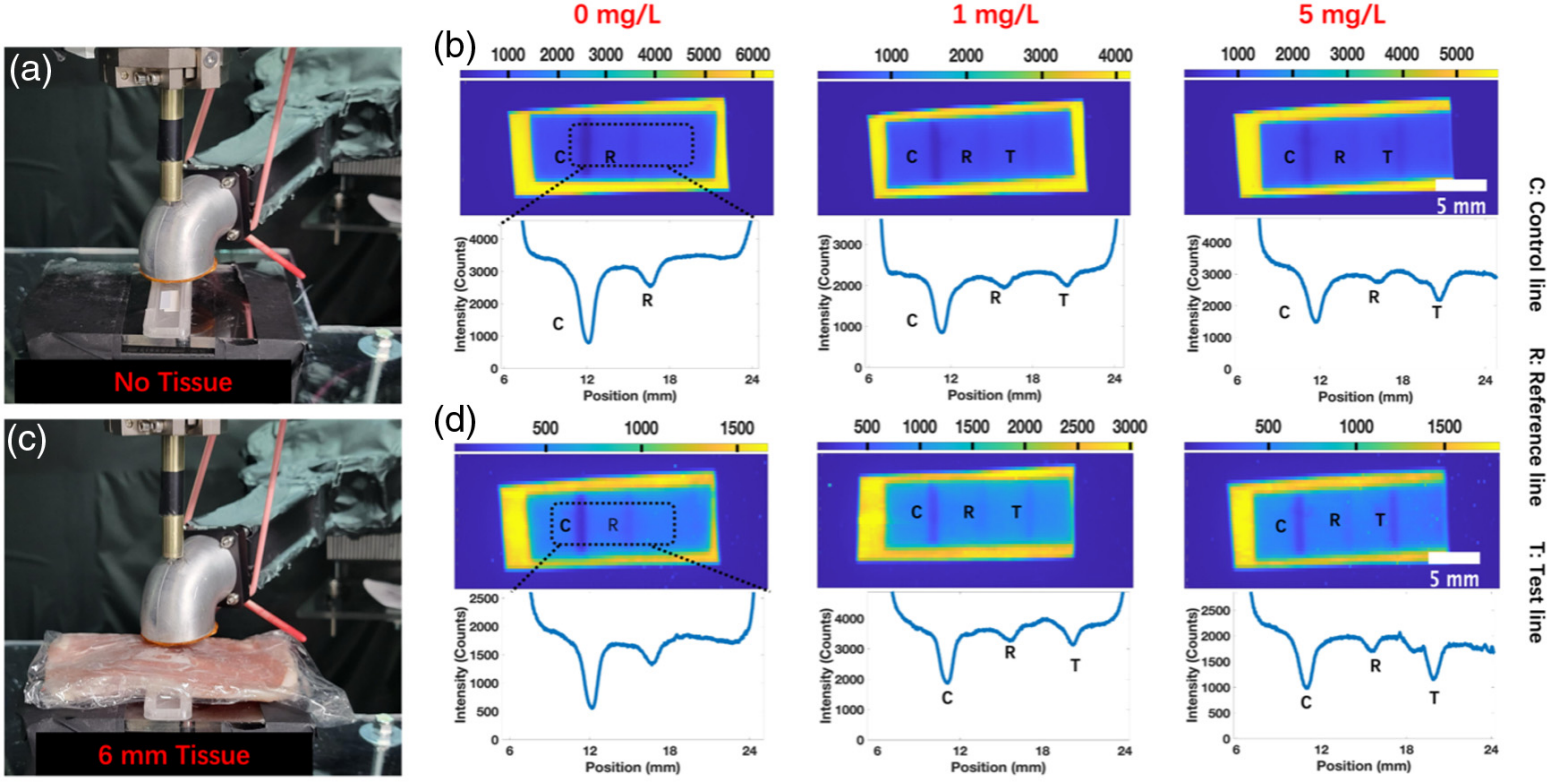
CRP assay setup and the XELCI images. (a) Setup of the LFA with a scintillator layer placed on the XELCI stage. (b) XELCI images of the LFA with scintillator layer without tissue, and the MATLAB-processed plots. (c) Setup of the LFA with a scintillator layer covered by 6 mm thick porcine tissue. (d) XELCI images of the LFA with a scintillator layer with a 6-mm thick porcine tissue slice and the MATLAB-processed plots. Each assay was performed with n=1 trial per concentration.

Photos of the setup without tissue are shown in Fig. 4(a) and with 6 mm of porcine tissue in Fig. 4(c). Figures 4(b) and 4(d) show the XELCI images of LFAs developed with varying CRP concentration without tissue and through tissue, respectively. The corresponding line profiles are below the XELCI images. The positions of the control, test, and reference lines could be easily distinguished in all images, and the intensity of light passing through the test lines decreases with increasing CRP concentration. This is expected since higher antigen concentrations cause more binding of the light-absorbing nanoparticle labels. As expected, the signal intensity decreased due to light attenuation by the tissue, but the LFA lines were still clearly visible and quantifiable compared to adjacent regions with no lines.

**Fig. 5:**
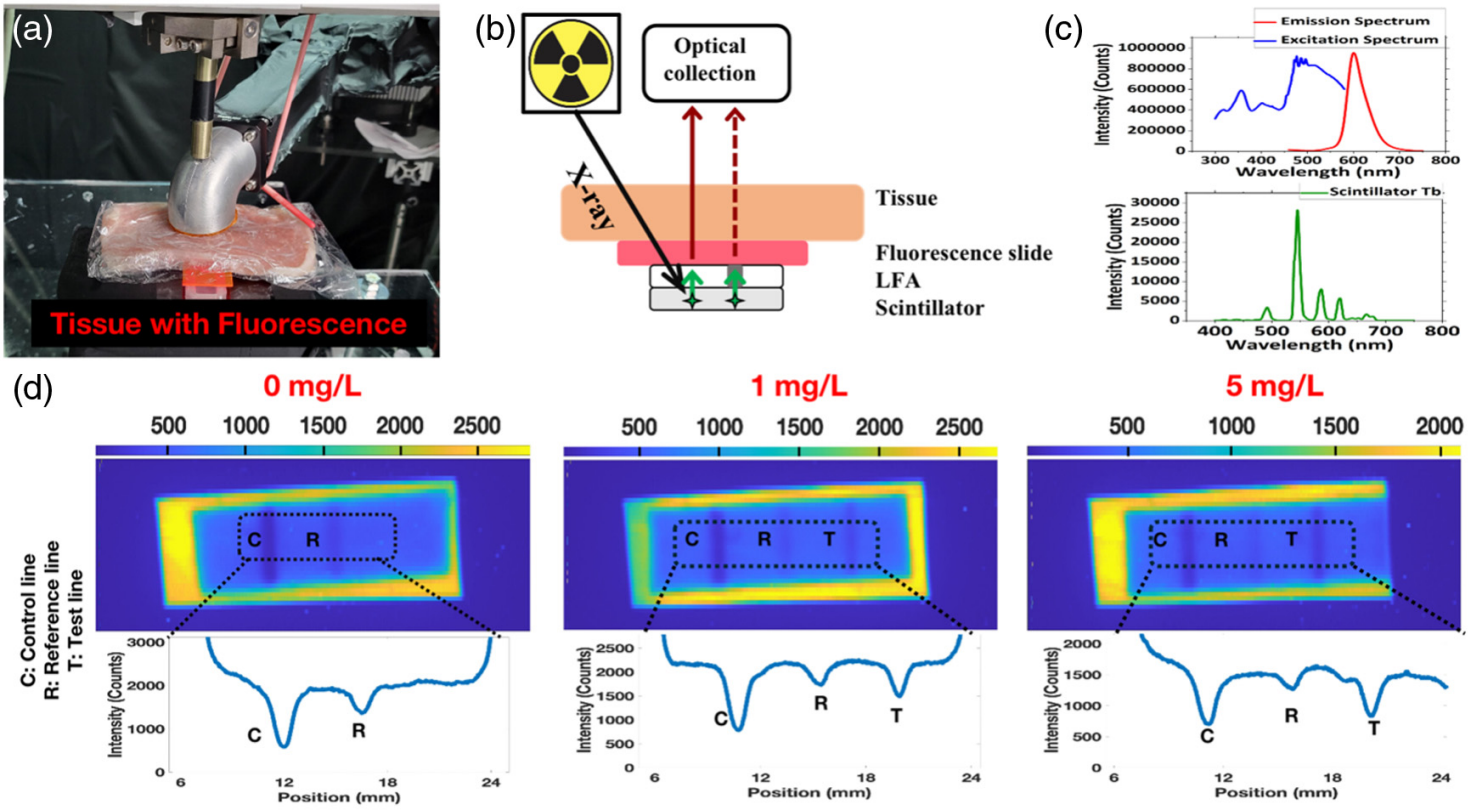
CRP fluorescence assay setup and the XELCI images. (a) Photo of LFA with scintillator layer and fluorescence slide covered by a 6-mm-thick porcine tissue. (b) Schematic of XELCI image with fluorescence slide. (c) Emission and Excitation spectra of the fluorescence slide and the emission spectrum of ${\mathrm{Gd}}_{2}O_{2}S:\mathrm{Tb}$ scintillator layer. (d) XELCI images of the LFA and scintillator layer combinations covered by fluorescence slide and 6-mm-thick porcine tissue. Each assay was performed with n=1 trial per concentration.

Under X-ray irradiation, the ${\mathrm{Gd}}_{2}O_{2}S:\mathrm{Tb}$ microphosphors emit predominantly green light [see Fig. 5(c)]. This green light is absorbed by the red gold nanoparticle labels but has limited penetration through thick tissue. Nonetheless, the lines were clearly visible on XELCI images through tissue, likely due to both gold nanoparticle absorption of secondary luminescence peaks at 620 and 680 nm, and some penetration through the 6-mm tissue of green light and green-excited tissue autofluorescence.

#### 3.2.1 Fluorescence

To increase the tissue penetration of the green radioluminescence from ${\mathrm{Gd}}_{2}O_{2}S:\mathrm{Tb}$, we inserted a fluorescent slide between the tissue and the LFA strips to convert the green radioluminescence passing through the LFA into red light (see Fig. 5). Figure 5(a) shows a photo of the assay setup, and Fig. 5(b) shows a schematic. The fluorescence slide excitation and emission spectra are shown in Fig. 5(c) and compared with the ${\mathrm{Gd}}_{2}O_{2}S:\mathrm{Tb}$ luminescence spectrum, which overlapped with the excitation spectrum. Figure 5(d) shows the XELCI images and the line profiles. A similar trend was observed with previous HCG and CRP experiments. The light intensity through tissue was higher than in studies without the fluorescent slide, indicating that converting the green light to more penetrating red light was effective.

#### 3.2.2 Spatial resolution

We note that the light spreads out as it scatters with a point spread function width typically larger than the depth of the tissue imaged through,^12^ but scanning our focused X-ray beam across the scintillator and measuring luminescence from each point enabled local absorption measurements with far greater resolution. In Figs. 2, 4, and 5, the images with and without tissue were comparable with each other, indicating that the spatial resolution was not strongly dependent on tissue. The XELCI images were also similar to the photographs without tissue, but somewhat less sharp, indicating some blurring in the XELCI images (primarily due to the finite X-ray beam width and scattering within the scintillator film).

We measured the control line peak width in the CRP assay (full width at half maximum intensity) with and without tissue. The width increased slightly in tissue (0.55 mm without tissue, 0.57 mm with tissue, and 0.58 mm with tissue and fluorescent slide). The nitrocellulose strip strongly scatters light, which both prevents incident scintillator radioluminescence from passing through to the tissue and prevents the fraction that does pass through from scattering back to the scintillator. Thus, the XELCI signal captured can be approximated as the transmittance through the strip times the transmittance through the tissue, with almost no contribution from light that passes through the film two or more times. Because the tissue is relatively uniform, especially on the 0.5-mm scale of the LFA lines, the image is minimally affected by the tissue except perhaps from some diffuse X-ray scattering. We measured the 80% to 20% knife edge for the edge of the nitrocellulose film and found 314  μm without tissue, 566  μm with tissue, and 574  μm with tissue and fluorescence (Fig. S8 in the Supplementary Material). Finally, we measured the 80% to 20% width of the edge of the scintillator film (where there was no nitrocellulose LFA strip) and found 157  μm without tissue, 306  μm with tissue, and 312  μm with tissue and fluorescence (Fig. S9 in the Supplementary Material). We attribute the increased width compared to the edge of the scintillator film to scattering in the thickness of the nitrocellulose strip. Although there was some broadening in the tissue, it was submillimeter in all cases, and least pronounced for reading the LFA assay, where the strip was face down with test, reference, and control lines touching the scintillator film.

#### 3.2.3 Data analysis

We also calculated the effective peak absorbance of the CRP assay for the control and test regions, using data across all components and concentrations. The effective absorbance was determined as the negative logarithm (−log10) of the ratio between the peak intensity and the adjacent background. Similar to the HCG analysis, the test-to-control (T/C) peak absorbance ratios for CRP exhibited strong correlations across different measurement conditions. Specifically, the correlation between T/C ratios obtained with and without tissue yielded an $R^{2}>0.99$, between without-tissue and fluorescence measurements yielded an $R^{2}>0.98$, and between with-tissue and fluorescence measurements yielded an $R^{2}>0.99$. All the plots were shown in Fig. S11 in the Supplementary Material.

The photographs of the CRP and HCG LFA strips reveal distinct colors, as shown in Fig. S12 in the Supplementary Material, making it difficult to visually determine which strip appears darker. In the XELCI images, the test-line dip in the HCG data was less pronounced than that in the CRP assay, suggesting differences in optical signal intensity between the two systems. This variation may stem from several factors: The commercial HCG and CRP LFA strips were prepared using different antibody-conjugated nanoparticles, which vary in particle color, size, concentration, and the width of the test and control lines, leading to differences in visual color intensity. In addition, the two scintillators exhibit different emission spectra and the nanoparticle labels have different absorbance spectra. Moreover, the optical setup differed between the two experiments: the HCG assay used a liquid light guide, whereas the CRP assay employed an acrylic light guide with an additional optical filter, which likely affected the light collection efficiency and contributed to the observed difference in signal contrast.

### 3.3 Proof-of-Principle for Storing LFAs in Wet Environments and Starting Flow with an External Trigger

LFA runs automatically when exposed to solvent, and the measurements are irreversible; thus, it is essential to keep the sample pad dry until the desired time to run the assay. For an implanted device, this requires an externally controllable valve or seal. To show proof of principle, we sealed an HCG LFA in parafilm and low-melting-point paraffin wax. This prevented the assay from running even when immersed in water. Melting the wax with an ultrasound beam exposed the sample pad to the solution, which ran the assay (Fig. S2 in the Supplementary Material). The results showed that we could target a specific location to run an assay with an external trigger. Admittedly, the ultrasound transducer was difficult to aim at the wax seal, required exposure for many seconds, and the 56°C melting point was above the damage threshold for surrounding tissue. Thus, in the future, other external triggers could be used for more precise targeting, including potentially running a series of different assays, each with a different programmable trigger via inductive, ultrasound, or other signals as previously developed for controlled drug release applications.^26^ Figure S10 in the Supplementary Material shows an example of how this might be implemented in future studies using an inductively coupled RF-key to specifically target one circuit to open a valve (e.g., either a wax microvalve^27^ or thermomechanical shape memory alloys valve^28^). The approach would require a power source, which could be based on a battery or an inductive power source. There are various types of implantable batteries, such as Mg-based biodegradable batteries,^29^ implantable Zn-based batteries,^30^ and lithium-based batteries fully encapsulated in polymer.^31^ These studies demonstrate the feasibility of the implanted power sources we will use in future research.

### 3.4 Limitations and Alternatives

#### 3.4.1 Device size and design

This study demonstrated proof of principle for detecting antigens through deep tissue using an X-ray addressed local light source. However, the prototype demonstration devices are not appropriate for implantation, and significant work would be needed to make them so. The prototype devices were not yet designed to fit on an implant. The size of the LFA and scintillator film combination is several square centimeters, which is too large for most implants. However, most of the area of the LFA is for the sample pad, absorptive pad, and a large region for observing the strip and background by eye, with only the 1-mm lines needed for sensing, reference, and test lines. Thus, we expect that the device could be dramatically miniaturized. For example, with 0.5-mm resolution imaging, we expect that 5 tests could be contained in a $1\;{\mathrm{cm}}^{2}\times 1\text{-}\mathrm{mm}$ thick device. A 5-mm long strip would be sufficient for a 1-mm reference region on each side of a test and sample line; with five 1-mm-width strips (or threads in shrink tubing)^32^ separated by 1 mm (1 cm total width) and additional pads, microvalves, and electronics stacked below, and with 50% extra for casing, etc. In addition, although the XELCI is useful for proof of principle, μ-LEDs could be used for measurements of specific regions without the need for an external X-ray and charged inductively^33^ or via ultrasound^34^ or near-infrared photovoltaics.^35^

#### 3.4.2 Imaging depth

We only imaged the device through 6 mm of tissue, which is sufficient for many applications in a mouse or superficial implant in a larger animal, but other applications require deeper implantation. We have previously shown that XELCI can image pH through 2 cm of the bone and soft tissue in an infected rabbit model, and this depth is useful for a wide range of human^12^ and animal devices.^8^ However, there is a trade-off between tissue thickness and signal intensity, with signal falling by a factor of 10 to 100 per centimeter of tissue.^36^ Though thicker tissue, one needs either to accept large X-ray doses, large pixel sizes, or poor spatial resolution. There is no sharp cutoff, but for most applications, we expect up to 2 cm would be feasible, but 5 cm would not. With higher power and the use of μ-LEDS, even 10 cm would also be feasible.^37^

#### 3.4.3 Biocompatibility

The approach requires that a device be implanted in the tissue. The surgical implantation is necessarily invasive, although with an integrated sensor, no additional surgery is needed and the measurement itself is noninvasive. Most medical implants are permanently left in place, unless there is a compelling reason to remove them; thus, long-term biocompatibility issues must be considered and justified long after sensor expiration. An exception is temporary antibiotic-impregnated joint spacers used to treat an infected prosthetic joint, which are typically removed and replaced with a permanent device within 6 months. Regardless, biocompatibility is needed for all implanted materials. The main components of the LFA are generally considered biocompatible although they are not optimized for implantation, and other materials could be substituted if needed: the casing is typically made of ABS, which is compatible with dental pulp stem cells,^38^ although Polyether ether ketone (PEEK) and polycarbonate urethane (PCU) could be used instead and are common for orthopedic implants; pads are typically made of cellulose which is generally considered biocompatible and is used to coat some hernia meshes; the strip is made of nitrocellulose which can be used wound dressing products.^39^^,^^40^ The conjugate pad is typically pretreated with ∼5  μL of several reagents, including PBS, sucrose, BSA, and Tween-20. Although some of these components are biocompatible with the human body, others will be eliminated during later processing steps.^41^ The gold nanoparticles and antibodies are generally considered biocompatible.^42^^,^^43^ The gold content in typical LFA strips is calculated to be ∼0.15 to 1.25  μg for 30 to 60 nm particles,^44^ giving doses of $10^{-8}\;g/\mathrm{kg}$ if given all at once to a human, which is orders of magnitude lower than reported toxic levels (e.g., $3.2\;g\;\mathrm{Au}\;{\mathrm{kg}}^{-1}\;{\mathrm{LD}}_{50}$ in a rat and no change in body weight for mice given $3\;\mathrm{mg}\;{\mathrm{kg}}^{-1}$ doses of 6.2 to 61.2 nm gold nanoparticles throughout the study period).^45^^,^^46^ In principle, the device could also be designed to self-seal after use, for example, using a polymer bridge within the PDMS that dissolves in time after exposure to fluid.^47^^,^^48^

#### 3.4.4 Single-use measurements

The LFA assay approach is single-use per strip and will not provide continuous measurements. Nonetheless, other designs can be envisioned where the nanoparticle labels are confined in a dialysis membrane, whereas the analyte can diffuse through; for such a system, lower $k_{\text{off}}$ antibodies (e.g., dissociation time constant <∼12  h) would be needed to have a reasonable daily measurement. This will be the subject of future research. Even here, the microvalve approach could preserve the antibodies in a test region in a dry state before use because LFAs typically last months at 37°C.

#### 3.4.5 Number of trials

A limitation of this study is that only one sample was tested per concentration (n=1). Multiple measurements would be needed at each concentration to analyze assay-to-assay variation and rigorously estimate detection limits. Nonetheless, the results for six HCG assay concentrations with and without tissue, and three for the CRP concentrations with and without tissue (and with versus without a fluorescent film) were sufficient to show proof of principle that proteins could be detected through tissue (e.g., Figs 2, 4, and 5 and Figs. S4–S9 in the Supplementary Material), and a high concordance between line profile measurements with versus without tissue (e.g., Fig. 3 and Fig. S11 in the Supplementary Material).

## 4 Conclusion

We constructed scintillator layers for CRP and HCG LFA strips and imaged them with XELCI. Placing tissue over the LFA strips obscured them from view due to optical scattering and absorption in the overlying tissue. However, XELCI images recovered the test and control lines, with only a small decrease in spatial resolution. The approach works for any optical absorption on the strip, including from Sharpie pen writing. In the future, we plan to develop arrays of sensors that can be implanted to monitor the presence of several analyte concentrations at chosen times for early detection and analysis of implant-associated infections.

## Supplementary Material

10.1117/1.JBO.30.S2.S23915.s01

## Data Availability

All data in support of the findings of this paper are available within the article or as supplementary material.
