# Supplementary material for: Local protein detection with lateral flow assay read through tissue using X-ray excited luminescence chemical imaging
Source: J Biomed Opt. 2025 Dec 10;30(Suppl 2):S23915. doi: 10.1117/1.JBO.30.S2.S23915 (PMC12694748; doi:10.1117/1.JBO.30.S2.S23915)
Supplement: Supplementary file 1 [file JBO_030_S23915_SD001.pdf]

Supplementary Information

Local Protein Detection with Lateral Flow Assay Read Through Tissue Using X-ray Excited Luminescence Chemical Imaging (XELCI)

Yu Ding,[a] Brad Kelly,[a,b] Morgan N. Reel[a], Matthew J. Case[a,c] and Jeffrey N. Anker [a,d]

[a] Department of Chemistry, Clemson University, Clemson, South Carolina 29634, United States
[b] Current address: Science Department, Green Upstate High School, Simpsonville, South Carolina 29681, United States;
[c] Current address: Department of Radiation Oncology, Emory University, Atlanta, Georgia 30322.
[d] Department of Bioengineering and Medical Biophysics Program, Clemson SC 29634

Table of Contents

Page

Figure S1.
Additional information about drilling hole on a cap of a non-digital Clear Blue pregnancy test, hole sealing using wax and the prepared non-digital Clear Blue pregnancy tests. 2

Figure S2.
Experimental setup of ultrasound and results of the experiment. 2

Figure S3.
Excitation spectrum of Gd_2O_2S:Eu scintillator films. 3

Figure S4.
Results of HCG LFA assay. 4

Figure S5.
Explanation of XELCI images and the corresponding line profile. 5

Figure S6.
All XELCI images and corresponding line profiles on CRP assay and the Excel plots of HCG assay. 6-8

Figure S7.
The calibration curves of HCG and CRP tests. 9

Figure S8.
Knife edge resolution with XELCI images of CRP LFA. 9

Figure S9.
Knife edge resolution with XELCI images of CRP LFA from another side. 10

Figure S10.
An example of implanted device using an inductively coupled IGM active filter and thermomechanical valves (melted wax with resistor) to control shape memory alloys. 11

Figure S11.
Correlation between **CRP** LFA data. 12

Figure S12
Photographs of CRP and HCG LFA 13

Table S1.
Example data from 1 mg/L XELCI image without tissue. 5

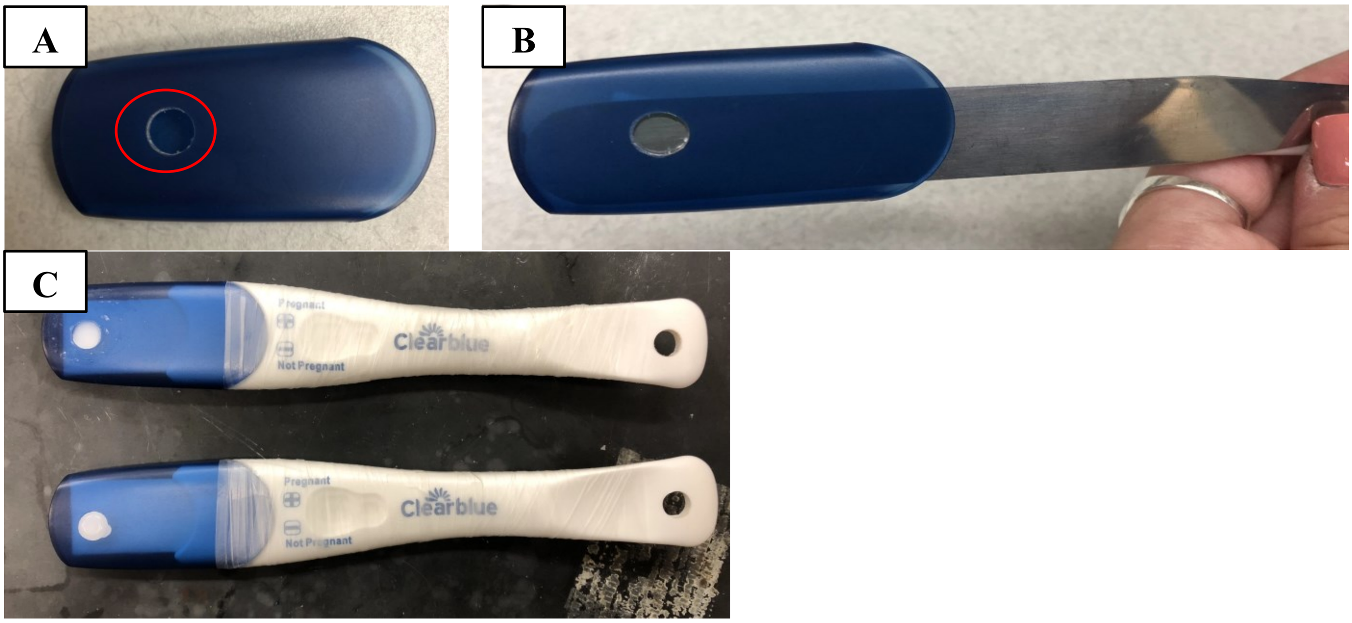

Figure S1. Modified hermetically sealed LFA with thermally-opened wax seal. **A)** A cap of a non-digital Clear Blue pregnancy test. A hole was drilled in the cap using the drill-bit machine. **B)** The cap of a non-digital Clear Blue pregnancy test with a hole drilled halfway through. A flat-sided tool was used as a barrier for the Paraffin wax when filling the drilled hole. **C)** Photo of two non-digital Clear Blur pregnancy tests with paraffin wax filling the drilled holes in the caps. The pregnancy tests were wrapped in Parafilm to prevent water from getting into the tests. The drilled holes at the base of the cap were made with a drill-bit machine.

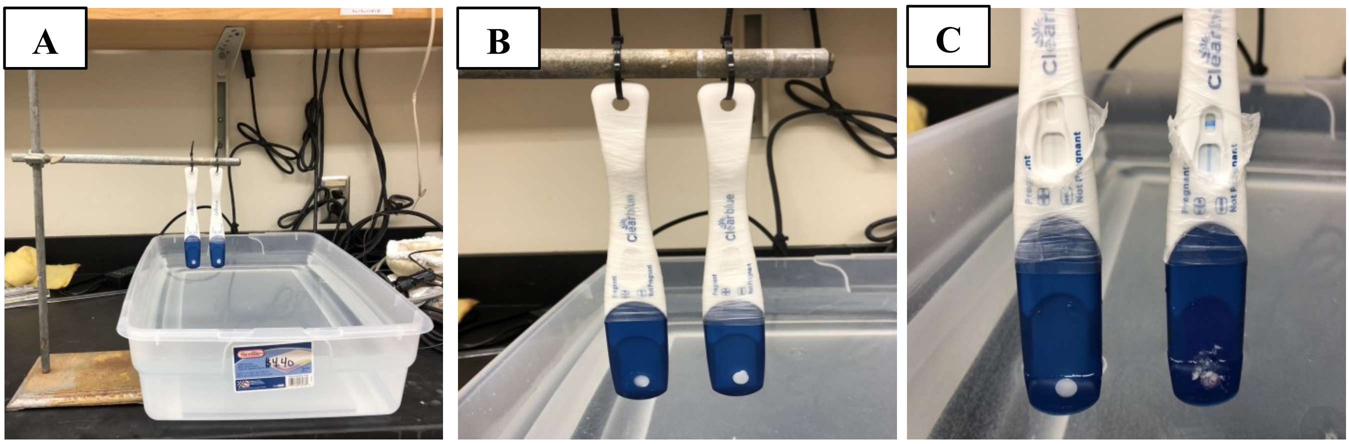

Figure S2. Photo of the experimental setup to show ultrasound controlled start time. **A)** A plastic bin was filled with water to ~2 inches from the top of the bin. A stand was used to hold the pregnancy tests in place while Zipties connected the tests to the extending rod. **B)** A close-up photo of the pregnancy tests connected to the extending rod by Zipties. The tests are not submerged in water in this image. **C)** Close-up photo of the LFAs after submerging in water. The wax seal on the left modified LFA is intact and the assay is blank, while the seal on the right was melted allowing the lateral flow assay to run resulting in a negative pregnancy test result.

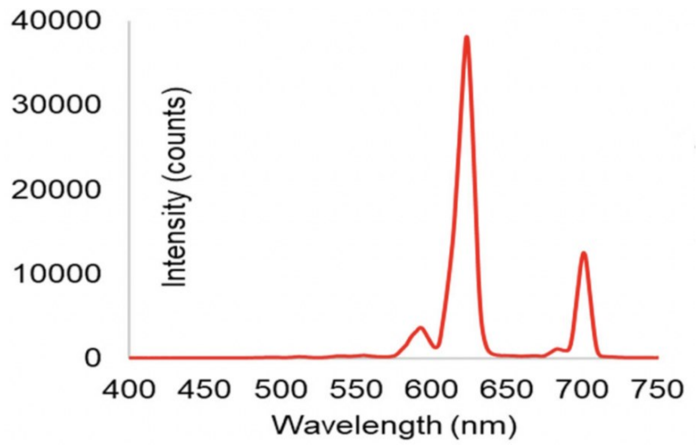

Figure S3. X-ray excited optical luminescence spectrum of Gd_2O_2S:Eu scintillator films.

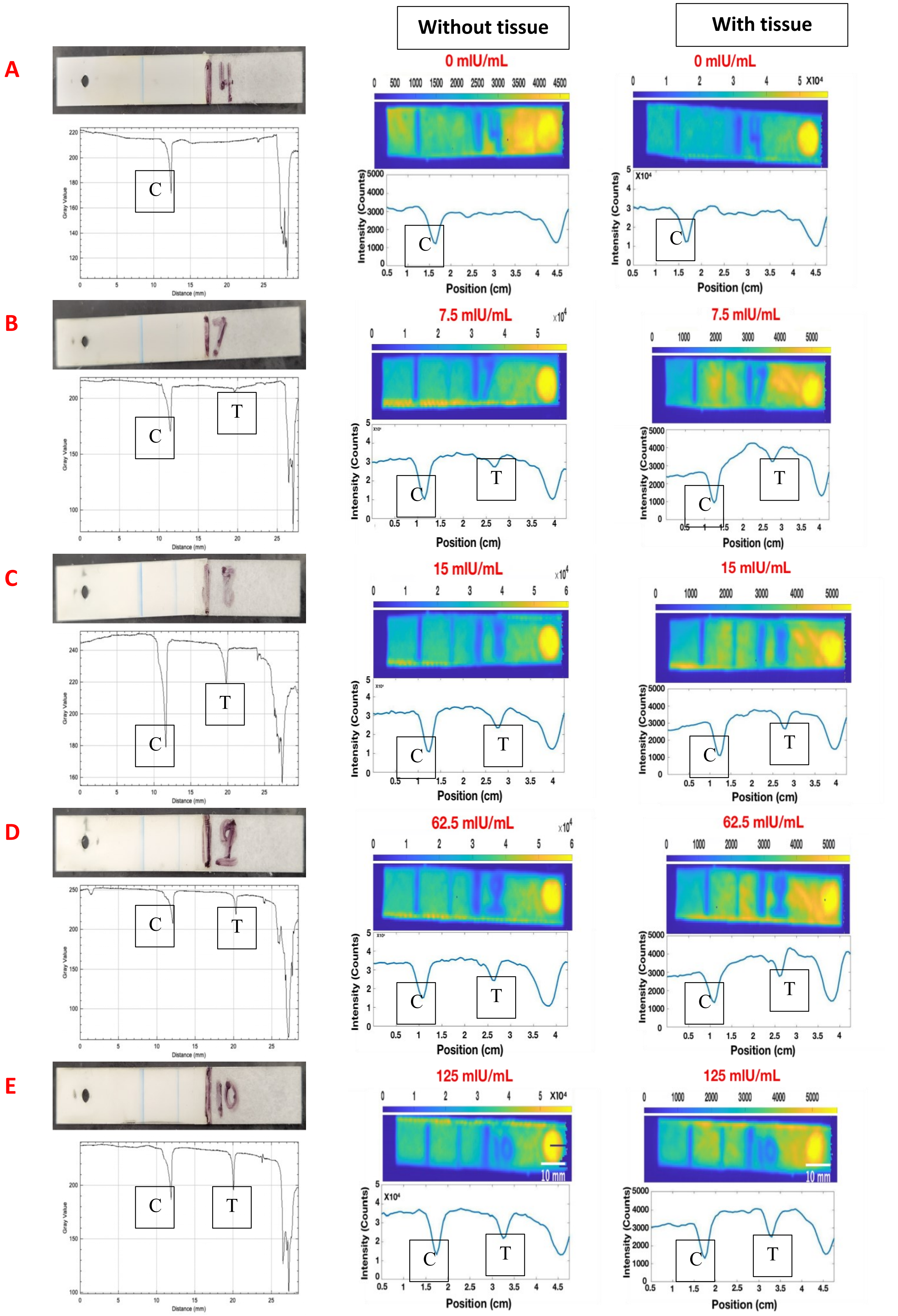

Figure S4. From left to right, photos of the HCG LFA strip and the intensity line profiles, XELCI images without tissue and corresponding line profile. A-E show tested HCG concentrations 0, 7.5, 15, 62.5, and 125 mIU/mL respectively.

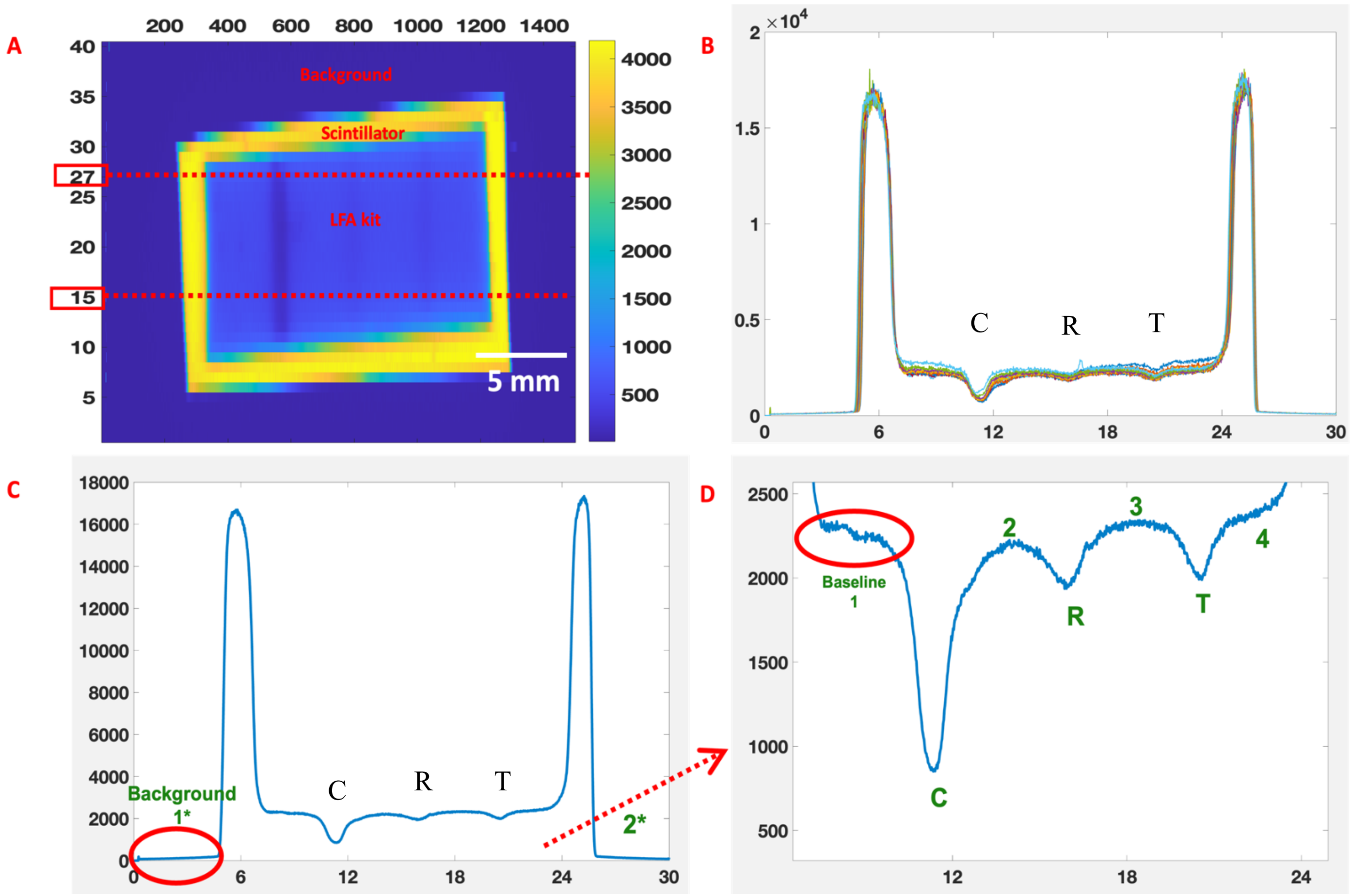

Figure S5. 1 mg/L, Without tissue example. **A)** The XELCI image of CRP LFA from PMT2. The central area surrounded by two dash lines need to do data analysis. The area is from line 15 to 27 in horizontal direction. **B)** Overall line scan plot from line 15 to 27. Each line has different color. **C)** Mean line profile from line 15 to 27. It contains two background regions labelled as 1* and 2*. **D)** Zoom in of the the LFA kit part to clearly observe the intensities and positions of Control, Reference and test lines.

	Data	Mean data
Background 1	109.5	108.4
Background 2	107.3	
Baseline 1	2256	2215
Baseline 2	2174	
Baseline 3	2292	2233
Baseline 4	2369	2331

Table 1. Example data from 1 mg/L XELCI image without tissue.

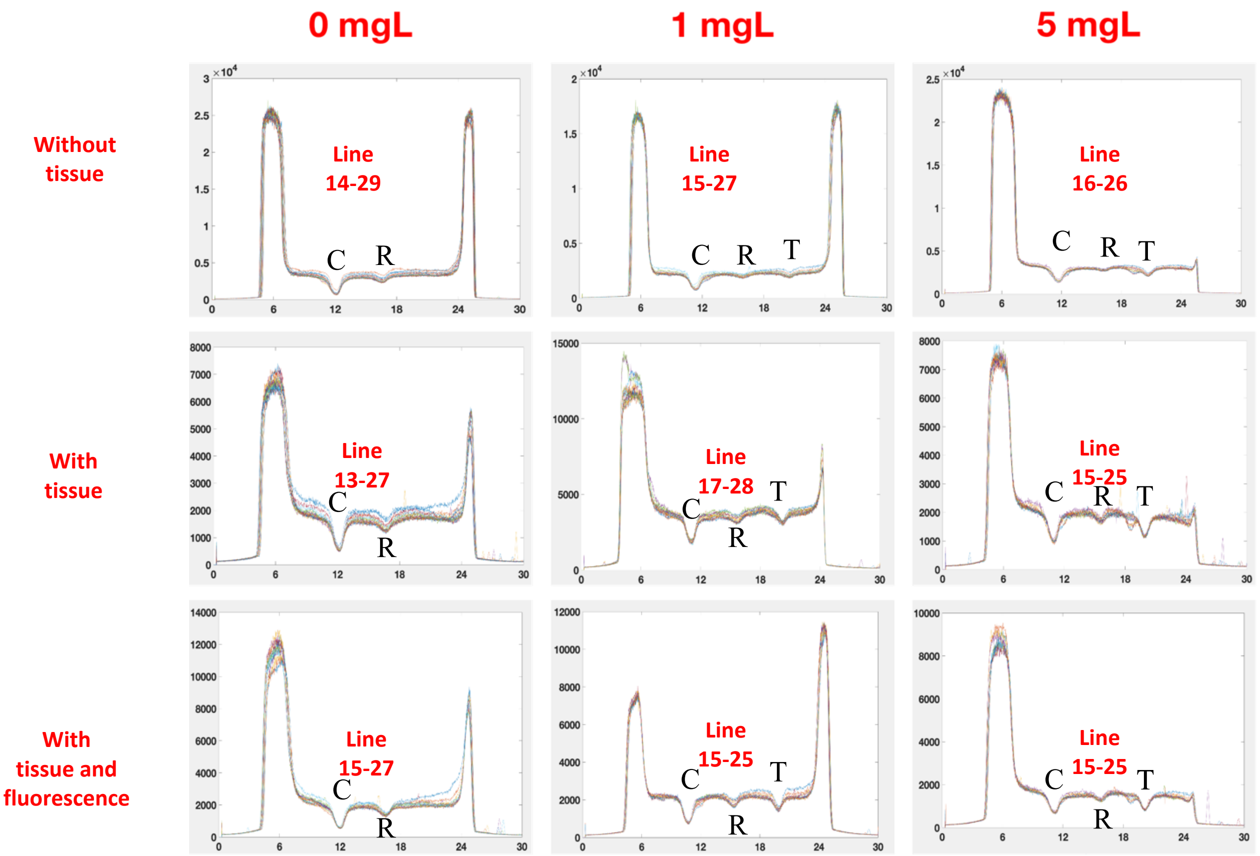

Figure S6 **A)** The MATLAB overall data plots of CRP test from 0 mg/L to 5 mg/L in three groups, without tissue, with tissue and with tissue and fluorescence. Each plot shows the line range.

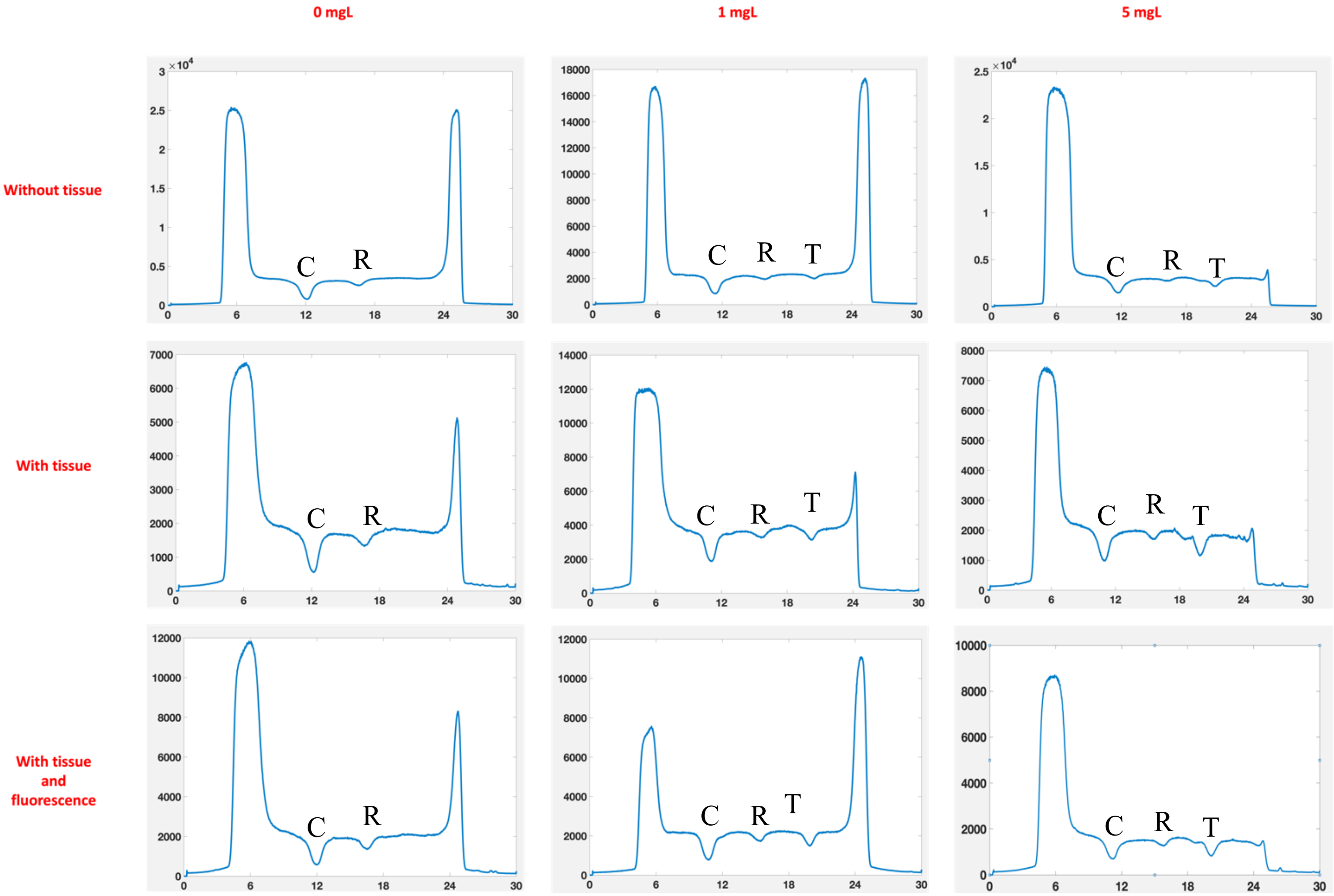

Figure S6 **B)** The MATLAB mean data plots of CRP test from 0 mg/L to 5 mg/L in three groups, without tissue, with tissue and with tissue and fluorescence.

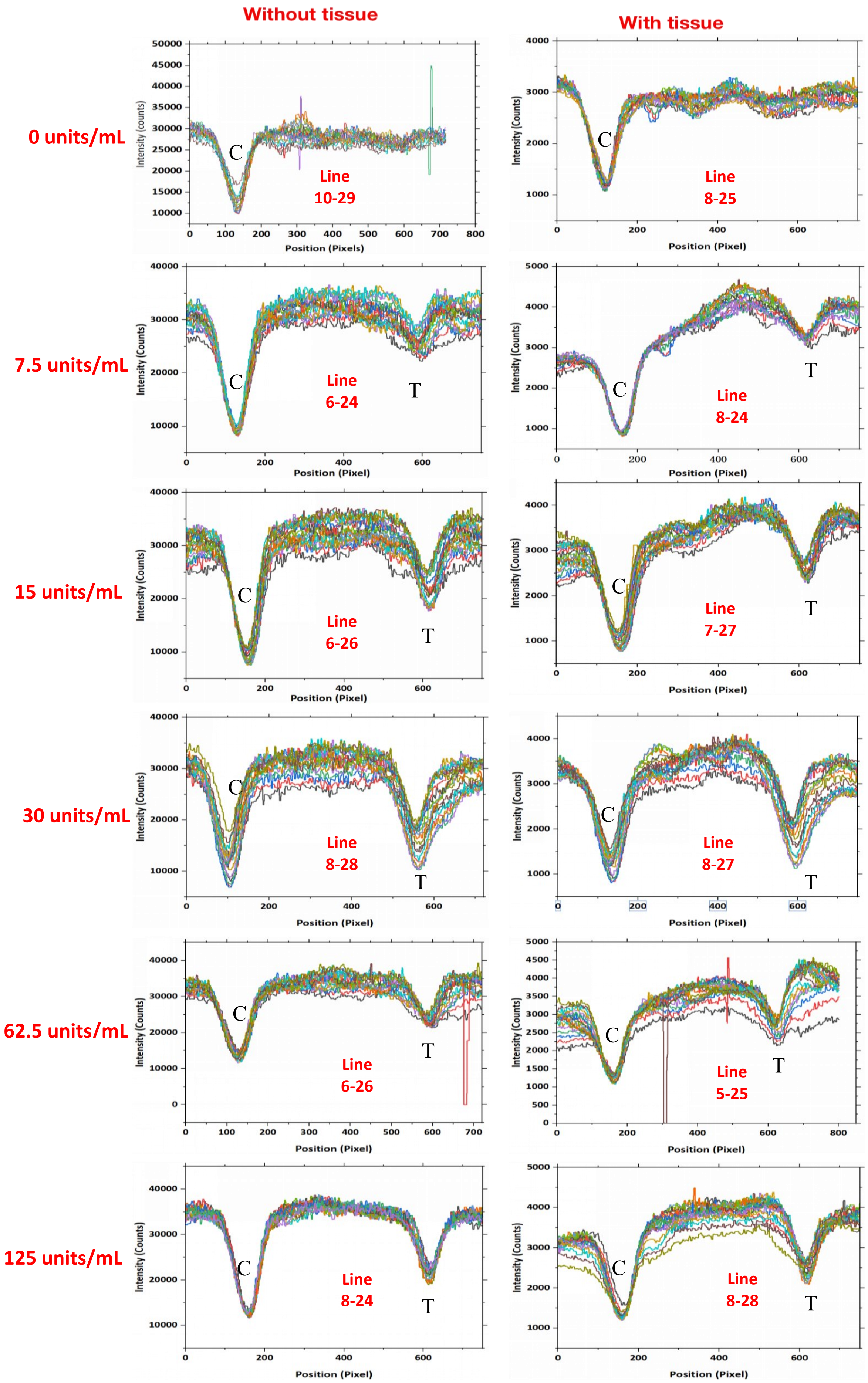

Figure S6 C) The overall data plots of HCG test from 0 units/mL to 125 units/mL in two groups, without tissue, and with tissue. Each plot shows the line range. Plots generated using Excel.

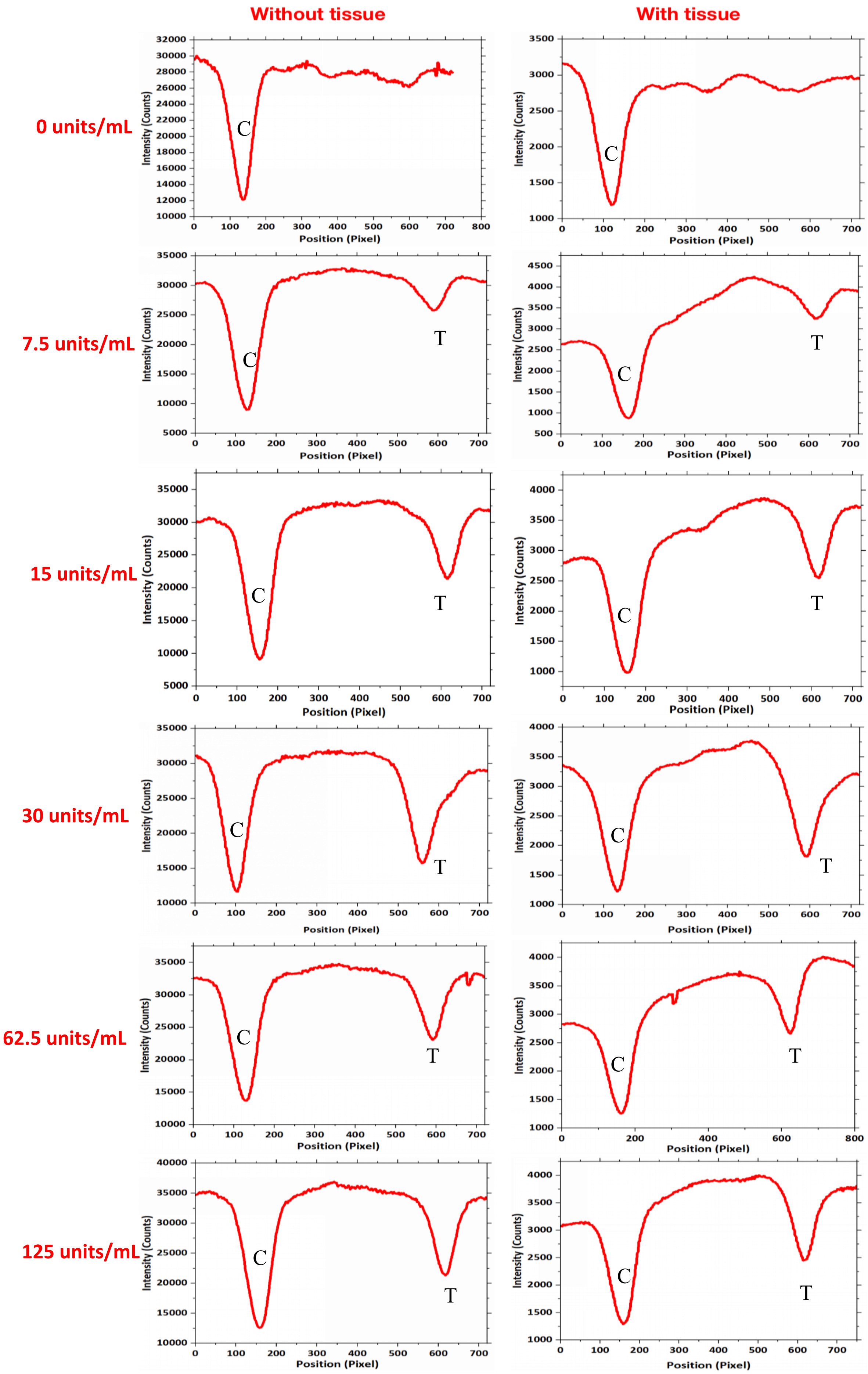

Figure S6 D) The Mean data plots of HCG test from 0 units/mL to 125 units/mL in two groups, without tissue, and with tissue. Plot generated using excel.

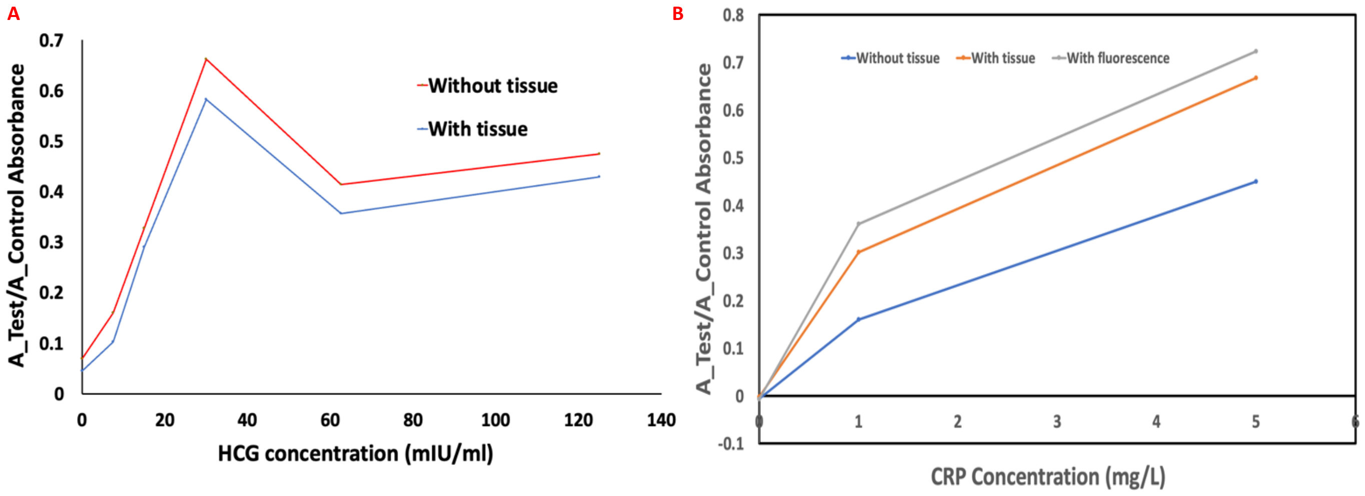

Figure S7. The calibration curves of HCG and CRP tests. A) The calibration curve of HCG test from concentration 0 mIU/mL to 125 mIU/mL. B) The calibration curve of CRP test from concentration 0 mg/L to 5 mg/L.

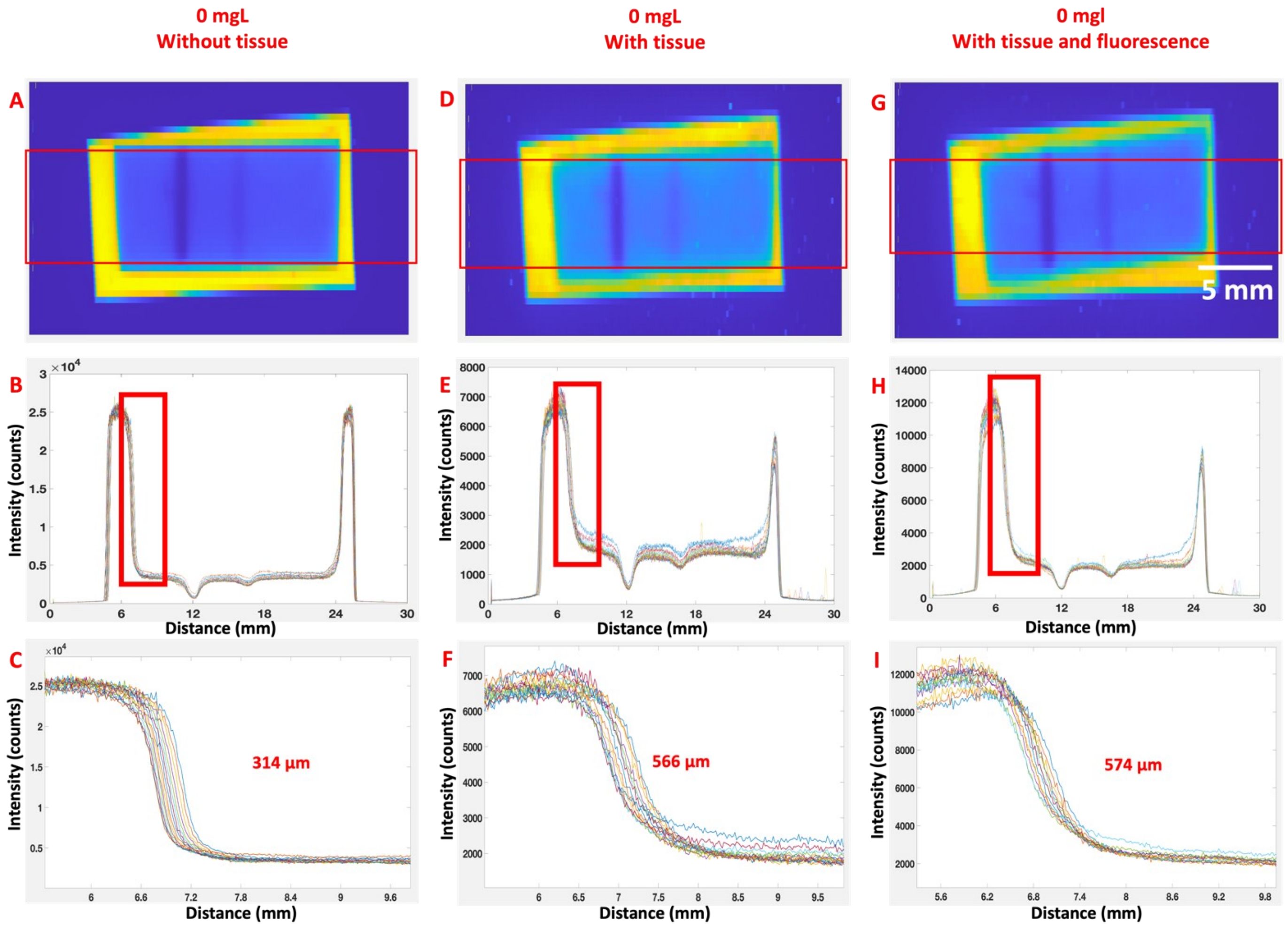

Figure S8. Measuring knife-edge resolution with XELCI images of CRP LFA based on the left edge of strip placed on the scintillator film. A) XELCI image of 0 mg/L, without tissue sample. B) Intensity plots of the red rectangle area from image (A). C) Zoom in of the red rectangle area from image (B) and used to calculate the 80%-20% knife edge resolution. D) XELCI image of 0 mg/L, with tissue sample. E) Intensity plots of the red rectangle area from image (D). F) Zoom in of the red rectangle area from image (E) and used to calculate the 80%-20% knife edge resolution. G) XELCI image of 0 mg/L, with tissue and fluorescence sample. H) Intensity plots of the red rectangle area from image (G). I) Zoom in of the red rectangle area from image (H) and used to calculate the 80%-20% knife edge resolution.

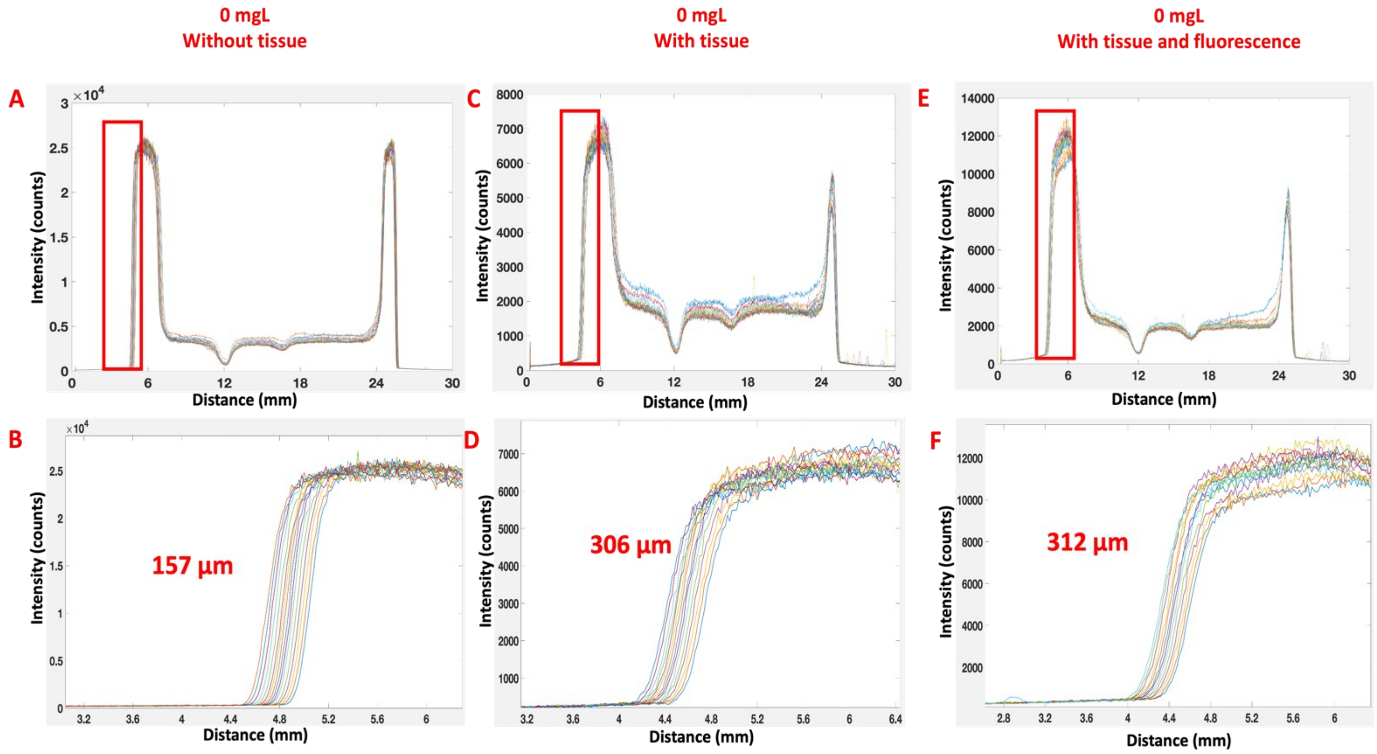

Figure S9. Measuring knife edge resolution with XELCI images of CRP LFA from left edge of the scintillator film. A) XELCI image of 0 mg/L, without tissue sample. B) Intensity plots of the red rectangle area from image (A) and used to calculate the 80%-20% knife edge resolution. C) XELCI image of 0 mg/L, with tissue sample. D) Intensity plots of the red rectangle area from image (C) and used to calculate the 80%-20% knife edge resolution. E) XELCI image of 0 mg/L, with tissue and fluorescence sample. F) Intensity plots of the red rectangle area from image (E) and used to calculate the 80%-20% knife edge resolution.

A A

B B

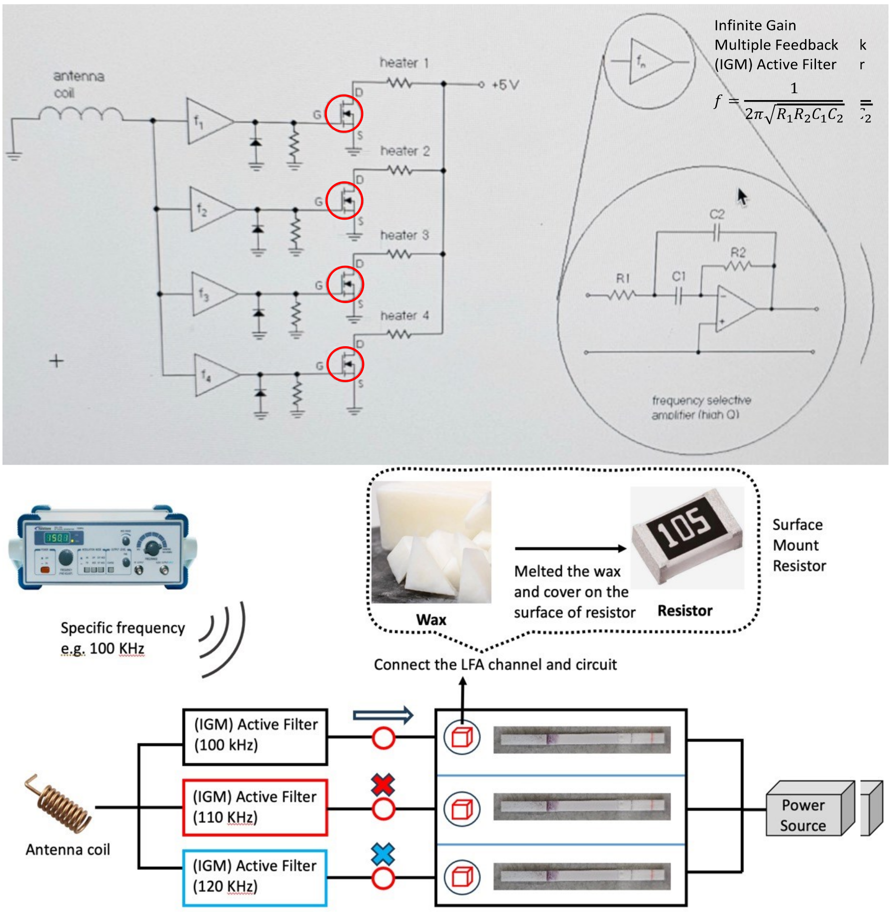

Figure S10. An example of implanted device using an inductively coupled IGM active filter and thermomechanical valves (melted wax with resistor or shape memory alloys) to open selected single-use sensor one at a time. **A)** Circuit Diagram of the system focusing on selecting inductive signal and filter to select a specific valve to open. **B)** Schematic of the whole system including power and sensor.

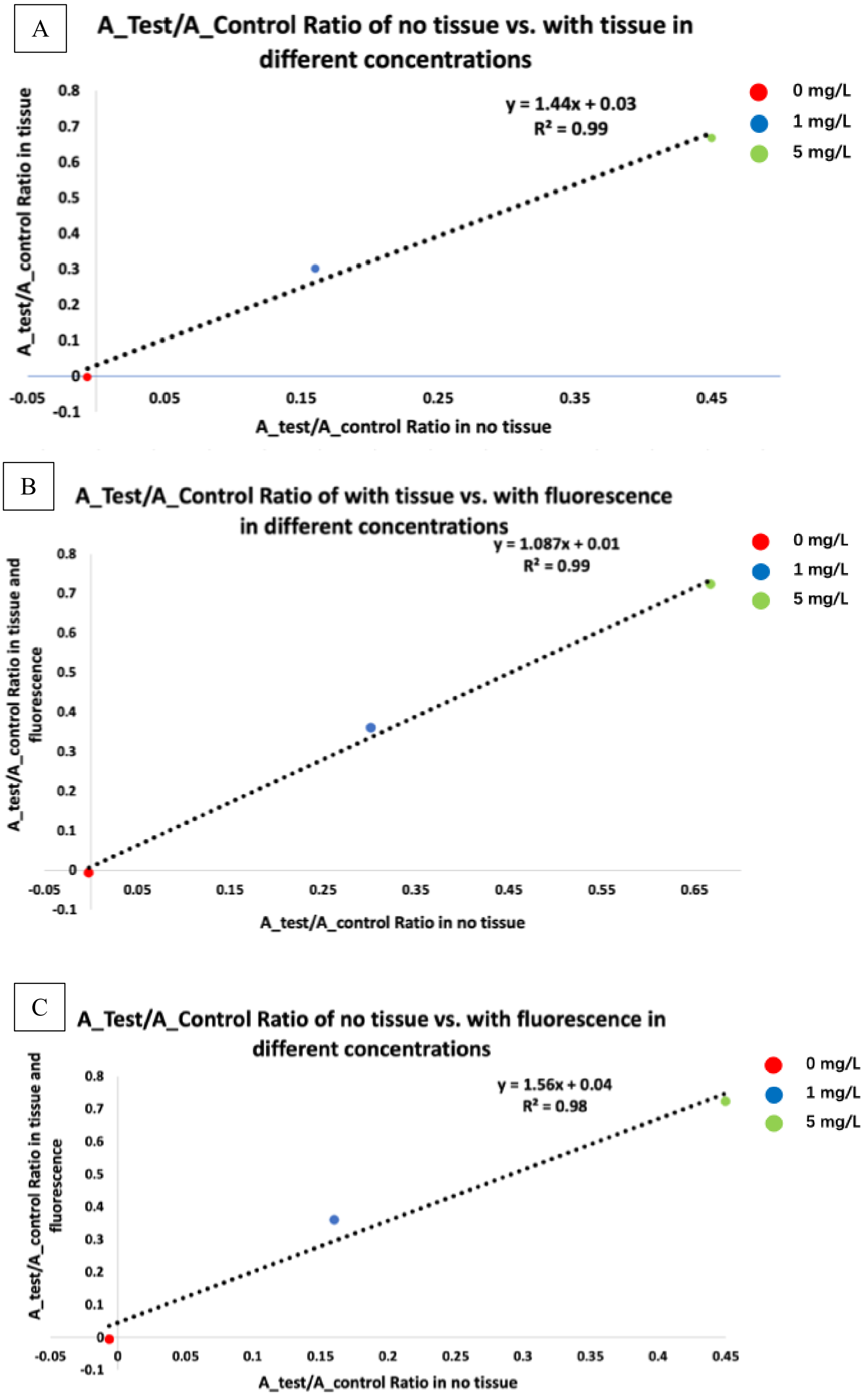

Figure S11: Correlation between CRP LFA data without tissue, with tissue and with fluorescence three situations. **A)** Ratio of Test line absorbance/ Control line absorbance with vs. without tissue. **B)** Ratio of Test line absorbance/ Control line absorbance with tissue vs. with tissue and fluorescence. **C)** Ratio of Test line absorbance/ Control line absorbance without vs. with tissue and fluorescence. All plots show strong agreement between measurements with and without tissue.

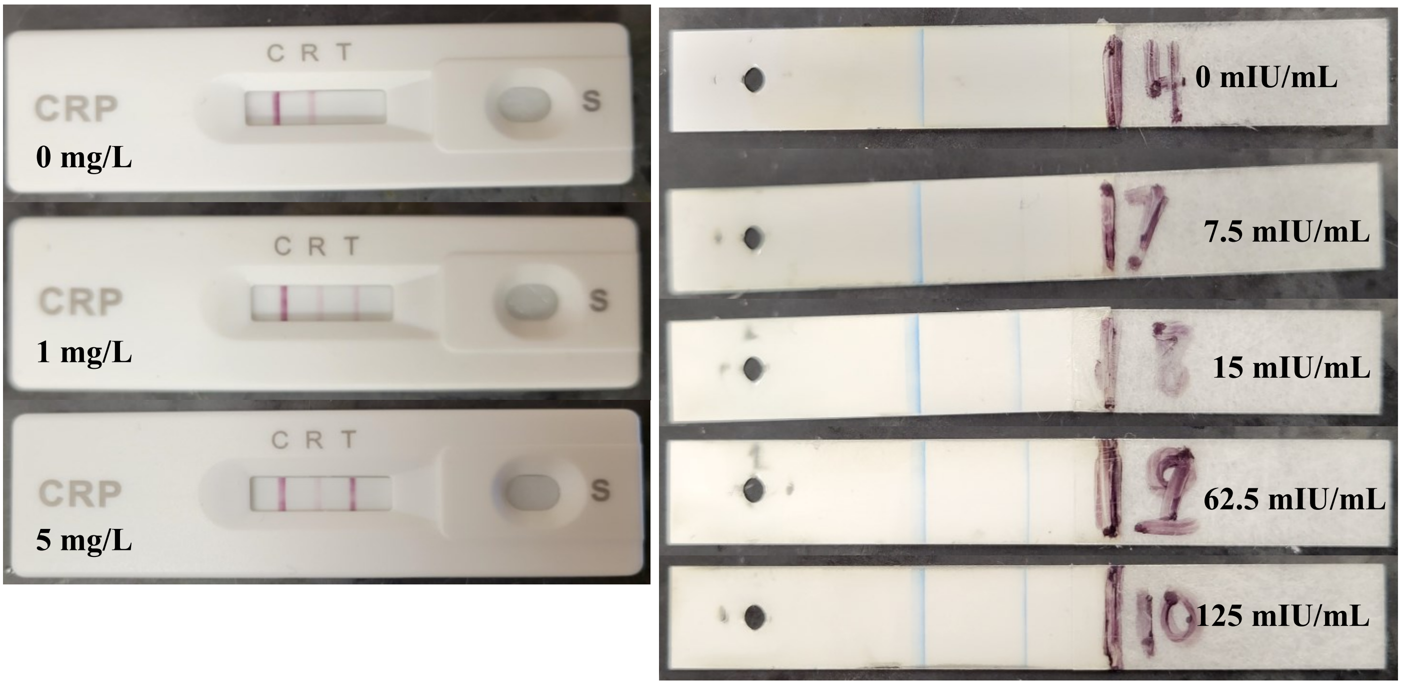

Figure S12: Photographs of CRP and HCG LFA strips developed with different analyte concentrations.
